# Synthesis, Structure and Molecular Docking of New 4,5-Dihydrothiazole Derivatives Based on 3,5-Dimethylpyrazole and Cytisine and Salsoline Alkaloids

**DOI:** 10.3390/molecules27217598

**Published:** 2022-11-05

**Authors:** Marat K. Ibrayev, Oralgazy A. Nurkenov, Zhanar B. Rakhimberlinova, Altynaray T. Takibayeva, Irina V. Palamarchuk, Dastan M. Turdybekov, Assel A. Kelmyalene, Ivan V. Kulakov

**Affiliations:** 1 Department of Chemistry and Chemical Technology, Abylkas Saginov Karaganda Technical University, Ave. Nursultan Nazarbayev, 56, Karaganda 100027, Kazakhstan; 2 Faculty of Chemistry, Karaganda Buketov University, st. University 28, Karaganda 100024, Kazakhstan; 3 Institute of Organic Synthesis and Coal Chemistry of Republic of Kazakhstan, Alikhanova 1, Karaganda 100008, Kazakhstan; 4 Institute of Chemistry, Tyumen State University, 15a Perekopskaya St., Tyumen 625003, Russia

**Keywords:** allylisothiocyanate, 1,2-dibromo-3-isothiocyanatopropane, 3,5-dimethyl-1*H*-pyrazole, intramolecular heterocyclization, 5-(bromomethyl)-2-(3,5-dimethyl-1*H*-pyrazol-1-yl)-4,5-dihydrothiazole, cytisine and salsoline alkaloids

## Abstract

The interaction results of 1,2-dibromo-3-isothiocyanatopropane with some pyrazoles as well as cytisine and salsoline alkaloids were presented in this paper. It was shown that the reaction resulted in one one-step and rather mild method for the preparation of the corresponding 1,3-thiazoline bromomethyl derivatives. The yield of this reaction was affected by the presence of a base and an order in which reagents were added. Molecular docking of the synthesized 1,3-thiazoline derivatives for putative antibacterial activity was carried out using the penicillin-binding target protein (PBP4) of the bacteria *E. coli* “Homo sapiens” and *S. aureus* “Homo sapiens” as an example. Molecular docking demonstrated that the compounds had insignificant binding energies at the level of selected reference drugs (*Cephalotin* and *Chloramphenicol*). The presence of natural alkaloids in the structure of thiazoline derivatives somewhat increased the affinity of these substrates for target proteins selected.

## 1. Introduction

The number of publications related to the synthesis and investigation of the biological activity of various thiazoles and their derivatives has recently increased. Thiazoles are five-membered heterocyclic compounds containing nitrogen and sulfur atoms. The thiazole itself exhibits high aromaticity. Hydrogenated thiazoles, namely 1,3-thiazoline and 1,3-thiazolidine, do not have aromatic properties. Hantzsch synthesis based on the reaction between α-haloketones (or α-haloaldehydes) with thioamides is one of the well-known methods for thiazole derivatives preparation [1,2,3,4]. To obtain dihydrothiazoles (thiazoline, thiazolidine) thiazole reduction methods are not applied, but intramolecular cyclizations of the corresponding thioamides are mainly put to use.

The thiazole cycle is widespread in natural compounds such as vitamin B1 (Thiamine), Penicillin, thiazole alkaloid, e.g., bacillamide A, B, C and neobacillamide A [5,6,7].

**Figure 1:**
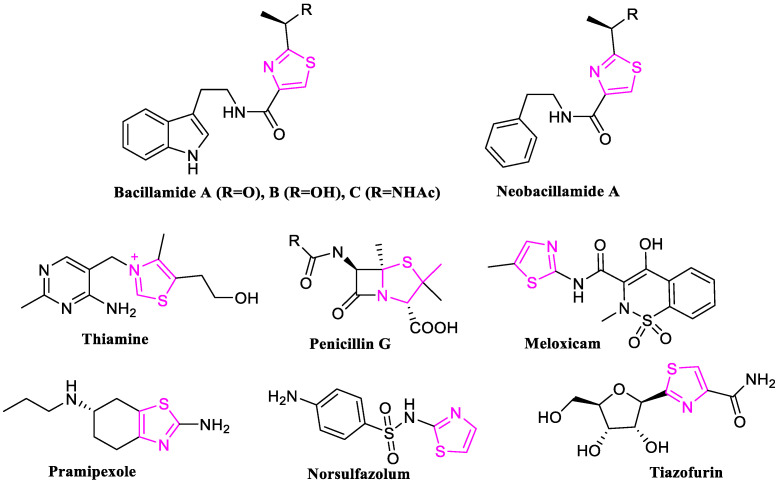
Natural and synthetic physiologically active compounds containing a thiazole cycle.

In addition, the thiazole scaffold is part of many drugs with high pharmacological activity, such as Meloxicam (non-steroidal anti-inflammatory drug) [8], Pramipexole (antidepressant) [9], Norsulfazolum (antimicrobial agent) [10], Tiazofurin (anticancer drug) [11] (Figure 1).

To date, there has been accumulated a huge amount of literature material on a wide range of biological activities of many other synthetic thiazole derivatives, such as antibacterial, antiviral, antifungal, anticancer, antiulcer, anti-inflammatory, antihypertensive, herbicidal, anthelmintic, antiparasitic, insecticidal ones [12,13,14,15,16,17,18,19,20,21,22].

Considerable interest in the synthesis of various thiazole derivatives is also associated with the discovery of their semiconductor properties, which determine their possible application in organic electronics as efficient solar cells and organic semiconductors [23,24].

Reduced forms of thiazoles, namely thiazolines have also received particular attention as preferred scaffolds with various pharmacological activities such as anti-inflammatory [25,26], antimicrobial [27,28,29,30], antidiabetic [31,32,33], anticancer [34,35,36] and antioxidant ones [37,38].

**Figure 2:**
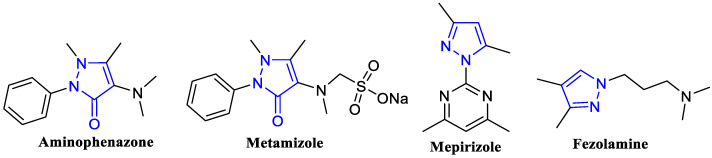
Pharmaceutical preparations containing a pyrazole heterocycle.

The combination of other physiologically active groups in the heterocyclic compounds structure can lead not only to an increase in the main therapeutic effect, but also to the emergence of new types of biological activity [39]. It is also known that many nitrogen-containing heterocyclic compounds are used in pharmaceutical practice as broad-spectrum drugs [40,41,42]. Pyrazole derivatives occupy a special place among them due to the greater preparative availability. In recent years, this has been caused by the increasing use of its derivatives as drugs, dyes, luminescent and fluorescent substances [43]. Pyrazole derivatives have proven to be effective pharmaceuticals with a variety of therapeutic activities, such as Mepirizole and Fezolamine (Figure 2), showing anti-inflammatory and antidepressant activities [44,45]. And, of course, among pyrazole derivatives, there are found substituted antipyrines (1-phenyl-2,3-dimethyl-5-pyrazolone) synthesized more than half a century ago, namely Aminophenazone and Metamizole, which exhibit antipyretic and analgesic effects [46,47] (Figure 2).

In works [48,49] the antidiabetic activity of new 1-substituted derivatives of 3,5-dimethylpyrazole was also demonstrated.

Quite often, halogenation methods and the introduction of sulfur-containing fragments are used as a chemical modification of many heterocyclic compounds. The presence of halogen substituents in the heterocycles structure results in the therapeutic effect amplification due to an increase in the lipophilicity of the resulting substances and facilitates their passage through biomembranes [50].

## 2. Results and Discussion

Most thiourea and thioureide derivatives have not only valuable pharmacological properties and are used as antiepileptic drugs [51], antidiabetic [52], anti-cancer [53], anti-tuberculosis [54] and other therapeutically active substances [55], but they are also initial synthons in the synthesis of many sulfur-containing heterocycles [56,57,58,59,60].

Isothiocyanate method [61] is one of the preparatively convenient methods allowing introducing thiourea or thioureide fragments into many aromatic and heterocyclic amines, including physiologically active alkaloids. It has been shown by us earlier on numerous examples [62,63,64,65].

The corresponding thioureas cyclization is used for the synthesis of many thiazole derivatives. Methods for the preparation of thiazoline heterocyclic derivatives from the corresponding allyl-containing thioureas under the action of various reagents, namely solutions of hydrogen halides and halogens, were described in the literature [66,67]. At the same time, compounds based on the pyrazole fragment, containing thiourea, thioamide or thiazole groups, are practically not described in the literature and represented by single examples [68].

Since interaction of isothiocyanates with amines was known to be the main method for obtaining thioureas, there was chosen 4-bromo-3,5-dimethylpyrazole **2** obtained by bromination of a preparatively available 3,5-dimethylpyrazole **1** to synthesize the corresponding thiourea derivative [69].

**Scheme 1:**
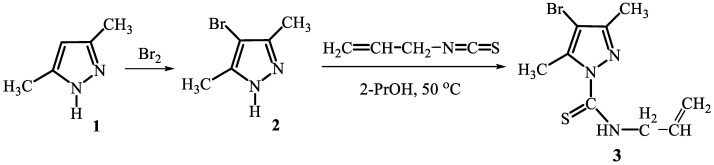
Synthesis of 4-bromo-3,5-dimethylpyrazole **2** and its allylthiourea derivative.

Further, on its basis, the interaction with allyl isothiocyanate was carried out (Scheme 1).

It was found that the reaction of 4-bromo-3,5-dimethylpyrazole **2** with the selected isothiocyanate, in contrast to simple and more basic alicyclic and aromatic amines, took longer time and required heating the reaction mixture to 50 °C. The yield of allylthiourea **3** was 71%. Analysis of the ^1^H NMR spectrum of compound **3** showed that there were intense narrow singlets of almost equivalent methyl protons of the pyrazole ring in the region of 2.08 and 2.15 ppm. The terminal allyl protons CH_2_ = of the thiourea fragment appeared as a system of multiplets from ddd and broadened dd in the region of 5.10–5.35 ppm with SSCC ^2^J = 17.0 Hz, ^3^J = 10.2 Hz. ^4^J = 1.5 Hz. The methine proton at the CH = double bond appeared as a complex multiplet (ddt) centered at 5.91 ppm. (Figure S1. Supplementary Material).

In the mass spectrum of N-allyl-(4-bromo-3,5-dimethyl-1*H*-pyrazole)-1-carbothioamide **3** with *m*/*z* and relative intensity *J_rel_* (%), in addition to two peaks of the molecular ion 273, 275 [M]^+^ (10.5%), there were fragments of the molecule decomposition along the N-C(S) bond with the basic 4-bromo-2,3-dimethylpyrazole fragment: 176, 174 (100%), as well as other fragments: 242, 240 (70%), 95 (39%), 41 (62%), 39 (90%) (Figure S7. Supporting Information), indicating the ease of the C–N bond cleavage between the pyrazole and thioamide fragments under an electron impact action.

**Scheme 2:**
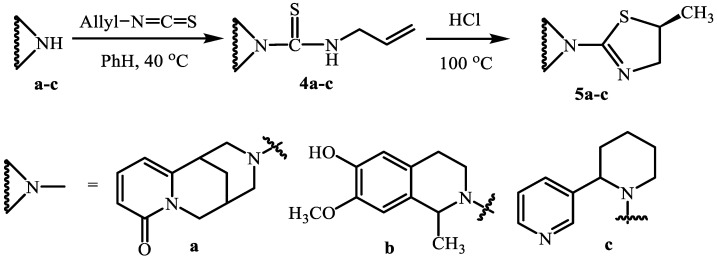
Synthesis of allylthiourea derivatives of alkaloids and cyclization.

In the works [70,71,72] it was shown that the corresponding N-allylthiocarbamides (**4a**–**c**) synthesized by the interaction of cytisine (**a**), salsoline (**b**), and anabasine (**c**) alkaloids with allylisothiocyanate in a solution of concentrated hydrochloric acid, when heated in a sealed ampoule for 5–6 h, underwent intramolecular cyclization to 1,3-thiazoline derivatives **5a**–**c** (Scheme 2).

**Scheme 3:**
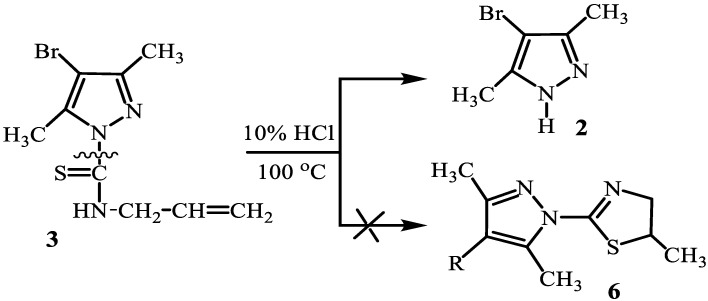
Acid hydrolysis of N-allyl-(4-bromo-3,5-dimethyl-1*H*-pyrazole)-1-carbothioamide **3**.

The following transformations in order to obtain new physiologically active compounds which combined the basic pyrazole ring and the sulfur-containing thiazole heterocycle in their structure were carried out. The ampoule cyclization of N-allyl-(4-bromo-3,5-dimethyl-1*H*-pyrazole)-1-carbothioamide **3** under the action of a hydrochloric acid solution was carried out by analogy with the procedures for N-allylthiocarbamides **4a**–**c** cyclization. However, when processing the reaction mixture, the expected corresponding cyclic 1,3-thiazoline **6** was not isolated, but the initial 4-bromo-3,5-dimethylpyrazole **2**, which was probably formed as a result of conventional hydrolysis at the C–N bond between the pyrazole ring and thioamide fragment (Scheme 3).

In order to obtain new 1,3-thiazoline pyrazole derivatives of type **6**, another alternative method for their preparation was used. The method was based on the use of a highly reactive isothiocyanate reagent, namely 2,3-dibromopropylisothiocyanate (**7**). The latter was used for a one-step synthesis of thiazoline derivatives via intramolecular heterocyclization of intermediate dibromo-substituted thioureas [73,74]. 2,3-Dibromopropylisothiocyanate (**7**) was synthesized by allylisothiocyanate bromination in chloroform medium according to the procedure [75].

**Scheme 4:**
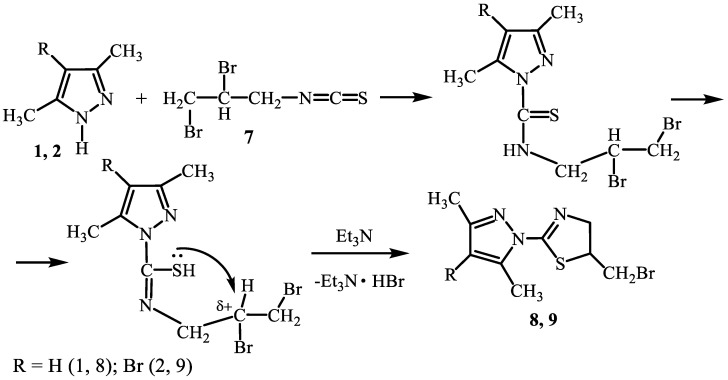
Mechanism of interaction of 2,3-dibromopropylisothiocyanate **7** with substituted pyrazole.

When carrying out the reaction of 3,5-dimethylpyrazole **1** and 4-bromo-3,5-dimethylpyrazole **2** with 2,3-dibromopropylisothiocyanate **7**, it was found that intramolecular heterocyclization of the intermediate 2,3-dibromopropyl-substituted thioureas occurred at Scheme 4:

It was found that the reaction course and the target products yield directly depended on the reagent’s addition order, the use of a solvent indifferent to the starting 2,3-dibromopropylisothiocyanate **7**, and a hydrogen bromide acceptor. So, for example, when adding a solution of 2,3-dibromopropylisothiocyanate **7** to an alcohol (or benzene) solution of 3,5-dimethylpyrazole **1**, about 50% of the original 3,5-dimethylpyrazole **1** remained unreacted at the reaction end, bound in the form of hydrobromide. It was possible to isolate the final reaction product in the form of a base in a high yield (96%) by changing the reagents addition order, using indifferent benzene and an excess of triethylamine as an acceptor of hydrogen bromide obtained during cyclization. 4-Bromo-3,5-dimethylpyrazole **2** resulted in the formation of the corresponding product **9** in a lower yield (65%).

The obtained bromomethyl 1,3-thiazoline derivatives of 3,5-dimethylpyrazole **8** and 4-bromo-3,5-dimethylpyrazole **9** are stable, well-crystallized white crystalline substances, soluble in many organic solvents and in hydrocarbons (when heated).

The composition, structure, and identity of the synthesized compounds **8**, **9** were confirmed by elemental analysis, IR, ^1^H NMR spectroscopy and mass spectrometry.

When analyzing the ^1^H and ^13^C NMR spectra of compounds **8**, **9** (Figures S2 and S3 Supporting Information) recorded in DMSO solution, it was found that a mixture of rotational isomers with axial and equatorial arrangement of the bromomethyl radical relative to the 1,3 planes of thiazoline ring was detected in the solution, which had been fixed by the corresponding duplicate peaks.

**Figure 3:**
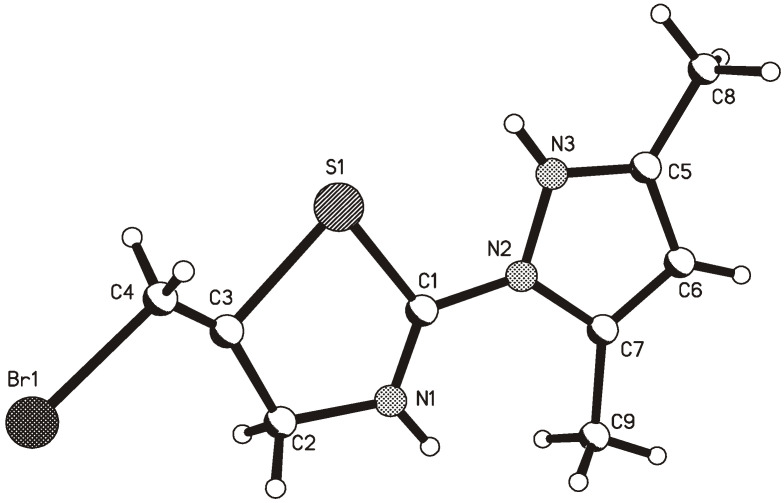
ORTEP representations of the compound **8** molecular structure.

X-ray diffraction investigation was carried out in order to establish the spatial structure of the 5-(bromomethyl)-2-(3,5-dimethyl-1*H*-pyrazol-1-yl)-4,5-dihydrothiazole molecule **8**. The molecule **8** general view is shown in Figure 3.

Analysis of structure **8** (CCDC 723563) showed that bond lengths and bond angles were close to usual [76]. The thiazoline ring adopted a strongly flattened sofa conformation (ΔC^8^_s_ = 1 Å). In our opinion, this was due to the strong ring tension, as well as the influence of a heavier bromine atom, which resulted in shortening the C4-C3 bond (0.997 Å) and elongating the C3-S1(2.049 Å) bond (Table S1. Supporting Information). Such anomalies are possible due to several factors, namely a molecule fragment disorder, possibly also during the cocrystallization of two isomers, or the crystal turned out to be an intergrowth (twin). The pyrazole cycle is planar with an accuracy of ±0.007 Å. The methyl groups are oriented equatorially and lie in the pyrazole ring plane.

**Scheme 5:**
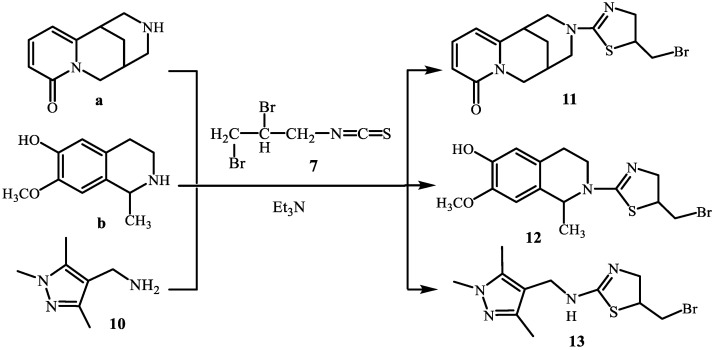
Interaction reaction of alkaloids (**a**), (**b**), amine **10** with 2,3-dibromopropylisothiocyanate **7**.

Thiazole derivatives that combine physiologically active alkaloids in their structure, namely cytisine and salsoline [77,78] which can potentially exhibit high pharmacological activity, are of particular interest. The reaction positive results prompted us to check the reproducibility of this reaction on the example of some biologically active alkaloids (cytisine **a**, salsoline **b**), as well as methylamino-substituted pyrazole (1,3,5-trimethyl-1*H*-pyrazol-4-yl)methanamine **10**, synthesized according to the method (Scheme 5).

As a result of the reactions performed, there were isolated in good yields (72–83%) the corresponding bromomethyl 1,3-thiazoline derivatives **11**–**13** based on physiologically active alkaloids, namely cytisine and salsoline, as well as methylamino-substituted pyrazole **10**. The resulting compounds **8**, **9**, **11**–**13** can be initial synthons for subsequent alkylation reactions and the synthesis of new biologically active compounds.

Structures of the synthesized compounds **11**–**13** were confirmed by elemental analysis, IR, ^1^H NMR spectroscopy and mass spectrometry.

When analyzing the ^1^H and ^13^C NMR spectra of compounds **11**–**13** (Figures S4 and S5. Supporting Information) recorded in a DMSO solution, as well as for compounds **8**, **9**, it was established that a mixture of rotational isomers with axial and equatorial arrangement of the bromomethyl radical relative to the plane of the 1,3-thiazoline ring was found in the solution.

The mass spectra analysis of compounds **11**–**13** showed that the molecules were not sufficiently stable under the electron impact action, since the spectra contained almost no peaks of the molecular ion, or they were present, but with a low relative intensity. All mass spectra showed intense fragments with eliminated hydrobromide [M-HBr] (Figures S9 and S10. Supporting Information).

### Molecular docking

According to numerous published data on the antibacterial activity of many thiazole derivatives, the molecular docking method was used to evaluate the possible putative antibacterial activity of the synthesized derivatives **8**, **9**, **11**–**13**. Penicillin binding protein 4 (PBP4) *E. coli* “Homo sapiens” (PDB: 2EXB) [79] and Penicillin binding protein 4 (PBP4) *S. aureus* “Homo sapiens” (pdb: 3HUN) [80] were chosen as target proteins.

Three-dimensional (3D) structures were obtained from the RCSB Protein Data Bank [81], while the ligand molecules were plotted with ChemBio3D Ultra 14.0. The protein structure was prepared for docking by removing the water molecule, the native ligand and adding polar hydrogen atoms, and converted to pdbqt format using the AutoDock MGL software package [82]. The docking process was carried out using AutoDock Vina [83]. Active site grid coordinates (X = 87.0, Y = −5.0 и Z = 45.0; the size 20 × 20 × 20 Å) were applied for (PBP4) *E. coli* “Homo sapiens” (PDB: 2EXB) [79] and active site grid coordinates (X = −22.0, Y = 4.0 и Z = 1.0; the size 20 × 20 × 20 Å) were applied for (PBP4) *S. aureus* “Homo sapiens” (pdb: 3HUN) [80]. Ligand interactions at binding sites were interpreted using the Discovery Studio Visualizer [84].

In addition, the structures of the standard antibiotics *Cephalotin* and *Chloramphenicol* were used as comparators due to their proven antibacterial activity [85,86].

**Figure 4:**
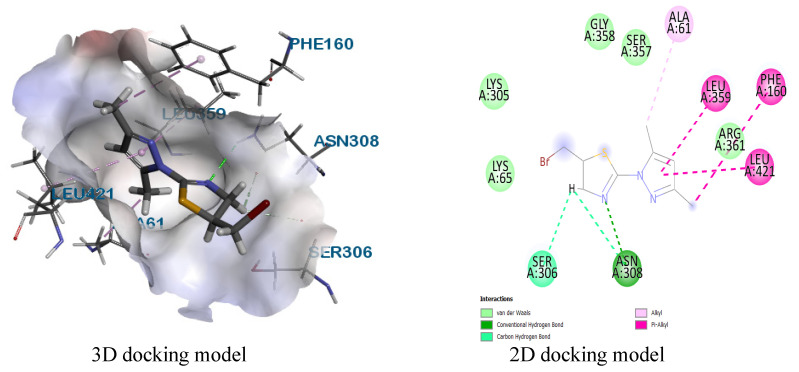
Complex between **8** and Penicillin binding protein 4 *E. coli* “Homo sapiens” (2EXB).

According to the molecular docking results, compound **8** had a weak binding energy of −5.2 kcal/mol with Penicillin binding protein 4 (PBP4) *E. coli* “Homo sapiens”, which was due to the formation of one hydrogen bond between the nitrogen atom of the thiazoline ring with the amino acid ASN 308 and two carbon-hydrogen bonds between the carbon atom of the thiazoline ring with amino acid SER 303 and the nitrogen atom of the thiazoline ring with amino acid ASN 308 (Figure 4).

**Figure 5:**
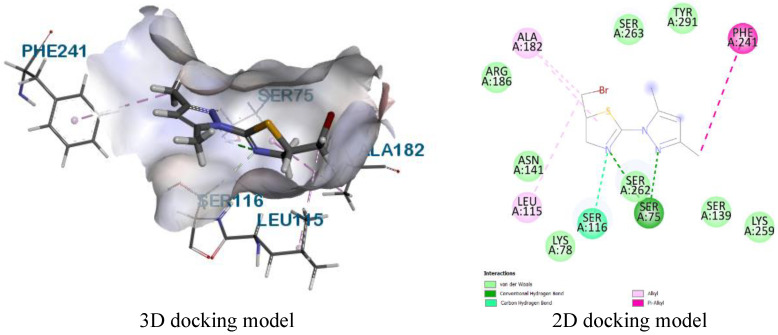
Complex between **8** and Penicillin binding protein 4 *S. aureus* “Homo sapiens” (3HUN).

Compound **8** had a weak binding energy of −5.6 kcal/mol with Penicillin binding protein 4 (PBP4) *S. aureus* “Homo sapiens”, which was associated with the formation of one carbon-hydrogen bond between the nitrogen atom of the thiazoline ring with the amino acid SER 116 and two hydrogen bonds between the nitrogen atoms of the thiazoline ring and the pyrazole ring with the amino acid SER 75 (Figure 5).

**Figure 6:**
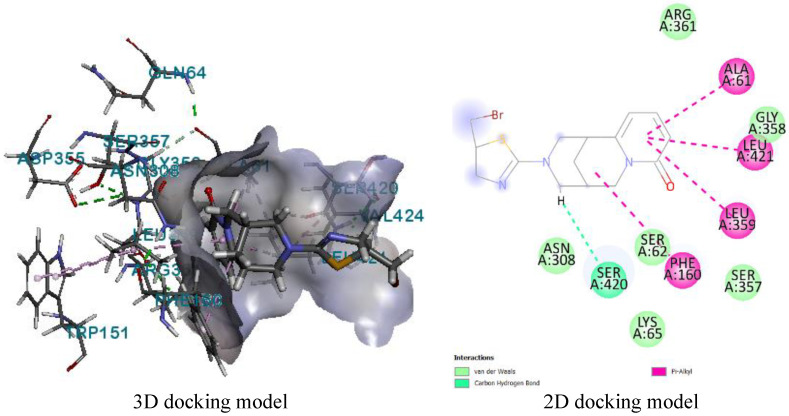
Complex between **11** and Penicillin binding protein 4 *E. coli* “Homo sapiens” (2EXB).

Derivative **11** showed a higher binding energy of −6.6 kcal/mol with Penicillin binding protein 4 (PBP4) *E. coli* “Homo sapiens” due to one carbon-hydrogen bond of the carbon atom (C-4) of the piperidine core of cytisine (Figure 6).

**Figure 7:**
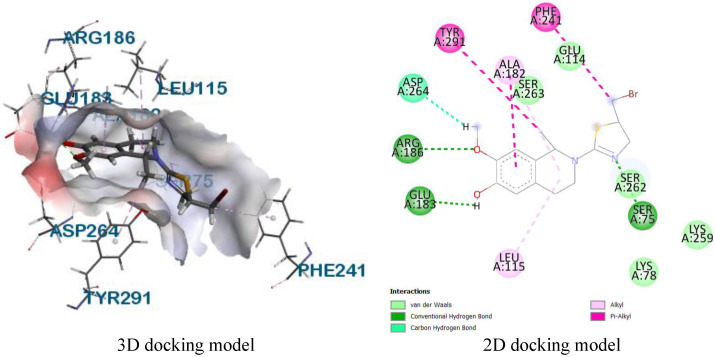
Complex between **12** and Penicillin binding protein 4 *S. aureus* “Homo sapiens” (3HUN).

Structure **12** had a higher binding energy of −7.5 kcal/mol with Penicillin binding protein 4 (PBP4) *S. aureus* “Homo sapiens” due to one carbon-hydrogen bond between the oxygen of the hydroxyl group with the amino acid ASP 264 and three hydrogen bonds between the oxygen of the hydroxyl group and methoxy groups of salsoline with amino acids ARG 186 and GLU 183 as well as between the nitrogen atom of the thiazoline ring with amino acid SER 75 (Figure 7).

Analysis of the interactions of 2EXB and 3HUN protein complexes with ligands **8**, **9**, **11**–**13** showed that all the studied derivatives formed sufficiently strong complexes with the target receptor protein (Table 1).

The number of intermolecular hydrogen bonds, the binding energy of stable ligand-receptor complexes (2EXB and 3HUN) and the number of the nearest amino acid residues were determined for the synthesized compounds **8**, **9**, **11**–**13** (Table 2).

## 3. Experimental

FTIR spectra were obtained with an Agilent Cary 630 spectrophotometer in a thin sample layer on a crystal attachment. ^1^H and ^13^C NMR spectra were recorded on a Bruker DRX400 (400 and 100 MHz, respectively) and Bruker AVANCE 500 (500 and 125 MHz, respectively) instruments using DMSO-*d_6_*, the internal standard was TMS or residual solvent signals (2.49 and 39.9 ppm ^1^H and for ^13^C nuclei in DMSO-*d_6_*).

Chromato-mass spectrometric studies were carried out on a Trace GC Ultra chromatograph with a DSQ II mass-selective detector in the electron ionization mode (70 eV) on a Thermo TR-5 MS quartz capillary column, 15 m long, 0.25 mm inner diameter, with a film thickness of the stationary phase of 0.25 μm. Splitless input mode was used. Carrier gas discharge was 20 mL/min. The velocity of the carrier gas (helium) was 1 mL/min. Evaporator temperature was 200 °C, transition chamber temperature was 200 °C, ion source temperature was 200 °C. The temperature of the column thermostat was changed according to the program, namely from 15 (5 min delay) to 220 °C at a rate of 20 °C per minute, to 290° at a rate of 15° per minute. The total analysis time was 30 min. The volume of the injected sample was 1 μL. Chromatograms were recorded in TIC mode. The range of mass scanning was 30–450 amu.

Melting points were determined using a Stuart SMP10 hot bench. Monitoring of the reaction course and the purity of the products was carried out by TLC on Sorbfil plates and visualized using iodine vapor or UV light.

**X-ray diffraction experiment.** Cell parameters and intensities of 1480 independent reflections were measured on a Bruker APEX-II CCD diffractometer, MoKα-radiation, graphite monochromator, θ/2θ-scan, 2θ ≤ 60°. There were rhombic crystals, a = 5.5149(5), b = 18.4636(7), c = 20.7277(11) Å, V = 2110.6(2)Å3, d_calc_ = 1.600g/cm^3^, Z = 8 (C_9_H_13_N_3_SBr), space group Pbca. The structure was elucidated by the direct method and refined by full-matrix least squares in the anisotropic approximation for non-hydrogen atoms. The H atoms were calculated geometrically and planted according to the “rider” type. The calculations used 958 reflections with I > 2σ(I). Final divergence factors were R = 0.082 and WR2 = 0.227. The structure was solved and refined using the programs “SHELXS-97” and “SHELXL-97”. The structure geometrical parameters were deposited with the CSDC (CCDC 723563).

### Experimental Procedures

N-allyl-4-bromo-3,5-dimethyl-1*H*-pyrazole-1-carbothioamide **3**. A solution of 1.75 g (0.01 mol) of 4-bromo-3,5-dimethylpyrazole in 10 mL of 2-propanol was added dropwise to a solution of 1 g (0.01 mol) of allylisothiocyanate in 10 mL of 2-propanol at a temperature of 50 °C. The solution was stirred for about 6 h. After the solvent distillation and recrystallization of the residue from hexane, there was obtained 1.95 g (71%) of a white crystalline substance with m.p. 50–52 °C. (2-PrOH). ^1^H NMR (500 MHz, DMSO-d_6_, δ, ppm, *J*/Hz): 2.08 (s, 3H, CH_3_); 2.15 (s, 3H, CH_3_), 4.22–4.37 (m, 2H, NH-CH_2_-CH=), 5.17 (ddd, 1H, *J* = 16.72, 10.3, 1.5, =C-H_a_), 5.29 (br. dd, 1H, *J* = 17.01, 10.2, =C-H_b_), 5.85–5.97 (ddt, 1H, *J* = 17.62, 10.25, 5.10, -CH=CH_a_H_b_), 12.58 (br. s, 1H, NH-CH_2_). ^13^C NMR (101 MHz, DMSO-d_6_) δ ppm 12.1, 12.7, 46.4, 98.0, 115.2, 135.1, 142.3, 148.2, 160.5. MS (EI) *m***/***z* (*I**_rel_***, %): 275 [M]^+^ (10), [M]^+^ 273 (10), 242 (69), 240 (71), 176 (100), 175 (78), 174 (99), 95 (39), 42 (46), 41 (62), 39 (89). Anal. calcd. for C_9_H_12_BrN_3_S: C, 39.78; H, 4.66; N, 15.81; found: C, 39.43; H, 4.41; N, 15.33.

5-(bromomethyl)-2-(3,5-dimethyl-1*H*-pyrazol-1-yl)-4,5-dihydrothiazole **8** (General method). A solution of 0.96 g (10 mmol) of 3,5-dimethylpyrazole **1** in 7 mL of benzene was added within 30 min to a solution of 2.60 g (10 mmol) of 2,3-dibromopropylisothiocyanate **7** and 1.01 g (20 mmol) of triethylamine in 10 mL of abs. benzene with vigorous stirring and a temperature of 20 °C. The solution was heated for about 3 h at a temperature of 40 °C. The precipitate of triethylamine hydrobromide was filtered off, washed with benzene. The benzene solution was evaporated to yield 2.63 g (96%) of a white crystalline substance with m.p. 101.5–102.5 °C (hexane-benzene). ^1^H NMR (500 MHz, DMSO-d_6_, δ, ppm, *J*/Hz): 2.15 (s, 3H, CH_3_); 2.47 (s, 3H, CH_3_), 3.65 (dd, 1 H, BrCH_2a_, *J* = 9.7, 9.7 Hz), 3.72 (dd, 1 H, BrCH_2b_, *J* = 9.9, 9.8 Hz), 4.25–4.30 (m, 3H, =N-CH_2_, S-CH), 6.15 (s, 1H, H-4 pyrazole). ^13^C NMR (125 MHz, DMSO-d6) δ ppm 13.1, 13.5, 37.0, 50.1, 65.2, 110.0, 142.1, 150.3, 156.1. Anal. calcd. for C_9_H_12_BrN_3_S: C, 39.81; H, 4.76; N, 15.52; found: C, 39.43; H, 4.41; N, 15.33.

2-(4-bromo-3,5-dimethyl-1*H*-pyrazol-1-yl)-5-(bromomethyl)-4,5-dihydrothiazole **9** was obtained by analogy with **8** from 2.60 g (10 mmol) of 2,3-dibromopropylisothiocyanate **7**, 1.01 g (20 mmol) of triethylamine and 1.75 g (10 mmol) of 4-bromo-3,5-dimethylpyrazole **2**. The yield was 2.29 g (65%); white crystals, m.p. was 106–107 °C (2-PrOH/hexane, 2:1). ^1^H NMR (400 MHz, DMSO-d_6_, δ, ppm, *J*/Hz): 2.16 (s, 3H, H_3_); 2.49 (s, 3H, CH_3_), 3.64–3.72 (m, 2 H, BrCH_2_), 4.28–4.33 (m, 3H, =N-CH_2_, S-CH). ^13^C NMR (101 MHz, DMSO-d_6_) δ ppm 12.1, 12.8, 37.0, 50.5, 65.2, 99.6, 139.9, 148.9, 156.1. MS (EI) *m***/***z* (*I**_rel_***, %): 355 [M]^+^ (5), [M]^+^ 353 (10), 351 [M]^+^ (5), 276 (5), 275 (12), 274 (95), 272 (100), 187 (15), 175 (78), 173 (90). Anal. calcd. for C_9_H_11_Br_2_N_3_S: C, 30.62; H, 3.14; N, 11.90; found: C, 30.94; H, 3.43; N, 12.27.

3-(5-(bromomethyl)-4,5-dihydrothiazol-2-yl)-1,2,3,4,5,6-hexahydro-8*H*-1,5-methanopyrido [1,2-a][1,5]diazocin-8-one **11** was obtained by analogy with **8** from 2.60 g (10 mmol) of 2,3-dibromopropylisothiocyanate **7**, 2.02 g (20 mmol) of triethylamine and 1.90 g (10 mmol) of cytisine **a**. The yield was 2.72 g (74%); white crystals, m.p. was 166–168 °C (2-PrOH/hexane 1:1). ^1^H NMR (400 MHz, DMSO-d_6_, δ, ppm, *J*/Hz): 1.97 (br. s, 2H, H-8), 2.56 (br. s, 1H, H-5), 3.16 (br. s, 1H, H-1), 3.24 (br. d, 2H, H-4*_a_*), 3.4 (d, 1 H, *J* = 5.0 Hz, H-2*_a_*), 3.42 (dd, 2H, BrCH_2_, *J*= 9.8, 6.2 Hz), 3.45 (m, 1H, H-4*_e_*) 3.70–3.80 (m, 2H, =N-CH_2_), 3.82–3.86 (m, 1H, H-2*_e_*), 3.98 (d, 1H, H-6*_a_*, *J* = 15.1 Hz), 4.02 (d, 1H, H-6*_e_*, *J =* 9.6 Hz), 4.08–4.14 (m, 1H, S-CH), 6.23 (d, 1H, H-11, *J*_11,10_ 6.9 Hz), 6.27 (d, 1H, H-9, ^3^*J*_9,10_ = 8.7 Hz), 7.38 (dd, 1H, H-10, ^3^*J*_10,5_ = 6.9 Hz, *J*_10,9_ = 8.7 Hz). ^13^C NMR (101 MHz, DMSO-d_6_) δ ppm 25.0, 26.9, 33.9, 36.3, 48.6, 52.5, 54.1, 55.6 62.9, 104.9, 115.8, 138.9, 149.9, 160.9, 162.1. MS (EI) *m***/***z* (*I**_rel_***, %): 288 (18), 287 (100), 160 (23), 146 (38), 141 (70), 113 (14), 100 (12). Anal. calcd. for C_15_H_18_BrN_3_OS: C, 48.92; H, 4.93; N, 11.41; found: C, 49.18; H, 5.17; N, 11.27.

2-(5-(bromomethyl)-4,5-dihydrothiazol-2-yl)-7-methoxy-1-methyl-1,2,3,4-tetrahydroisoquinolin-6-ol **12** was obtained by analogy with **8** from 2.60 g (10 mmol) of 2,3-dibromopropylisothiocyanate **7**, 2.02 g (20 mmol) of triethylamine and 1.93 g (10 mmol) of salsoline **b**. The yield was 3.08 g (83%); white crystals, m.p. was 149–150 °C (2-PrOH/hexane 1:1). ^1^H NMR (400 MHz, DMSO-d_6_, δ ppm): 1.37 (d, 3H, NCH-CH_3_, *J* = 6.4); 2.54 (dt, 1H, H-4_ax_, *J* = 16.0, 3.2 Hz), 2.71 (ddd, 1 H, *J* = 16.0, 10.8, 5.7 Hz, H-4_eq_), 3.34–3.42 (m, 2H, H-3), 3.53 (t, 1 H, BrCH_2a_, *J* = 9.2 Hz), 3.61 (dd, 1 H, BrCH_2b_, *J* = 10.1, 9.5 Hz), 3.72 (s, 3H, OCH_3_), 3.85–3.96 (m, 2H, =N-CH_2_), 4.16–4.22 (m, 1H, S-CH), 4.90 (q, 1 H, NCH-CH_3_, *J* = 6.3 Hz,), 6.50 (s, 1H, H-8), 6.70 (s, 1H, H-5), 8.77 (br. s, 1H, O-H). ^13^C NMR (101 MHz, DMSO-d_6_) δ ppm 21.6, 27.3, 36.5, 42.0, 52.3, 54.2, 55.8, 62.9, 110.5, 115.1, 125.4, 128.5, 145.0, 146.2, 159.5. MS (EI) *m***/***z* (*I**_rel_***, %): 372 [M]^+^ (10), [M]^+^ 273 (10), 242 (69), 240 (71), 176 (100), 175 (78), 174 (99), 95 (39), 42 (46), 41 (62), 39 (89). Anal. calcd. for C_15_H_19_BrN_2_O_2_S: C, 48.52; H, 5.16; N, 7.54; found: C, 48.71; H, 5.31; N, 7.73.

5-(bromomethyl)-N-((1,3,5-trimethyl-1*H*-pyrazol-4-yl)methyl)-4,5-dihydrothiazol-2-amine **13** was obtained by analogy with **8** from 2.60 g (10 mmol) of 2,3-dibromopropylisothiocyanate **7**, 2.02 g (20 mmol) of triethylamine and 1.39 g (10 mmol) of (1,3,5-trimethyl-1H-pyrazol-4-yl)methylamine **10**. The yield was 2.28 g (72%); white crystals, m.p. was 95–97 °C (PhH/hexane 1:1). ^1^H NMR (500 MHz, DMSO-d_6_, δ, ppm, *J*/Hz): 2.06 (s, 3H, CH_3_); 2.16 (s, 3H, CH_3_), 3.60 (s, 3H, CH_3_), 3.56–3.62 (m, 1 H, BrCH_2a_), 3.66 (dd, 1 H, BrCH_2b_, *J* = 9.9, 9.8 Hz), 3.87–3.93 (m, 2H, =N-CH_2_), 4.08, 4.12 (2 d, 2 H, HN-CH_2ab_, ^2^*J_ab_* = ^2^*J_ba_* = 14.3 Hz), 4.16–4.21 (m, 1H, S-CH), 7.37 (s, 1H, N-H). MS (EI) *m***/***z* (*I**_rel_***, %): 318 [M]^+^ (2), [M]^+^ 316 (2), 351 [M]^+^ (5), 138 (25), 123 (100), 122 (28), 56 (66), 55 (22), 41 (28), 39 (29). Anal. calcd. for C_11_H_17_BrN_4_S: C, 41.65; H, 5.40; N, 17.66; found: C, 41.17; H, 5.74; N, 17.91.

## 4. Conclusions

Thus, we found that an attempt to synthesize cyclic 1,3-thiazoline **6** by cyclization of the corresponding N-allyl-4-bromo-3,5-dimethyl-1*H*-pyrazole-1-carbothioamide **3** under the action of hydrochloric acid solution led to hydrolysis to the starting 4-bromo-3,5-dimethylpyrazole **2**. The interaction of 1,2-dibromo-3-isothiocyanatopropane with the indicated pyrazoles **1**,**2**,**10**, as well as cytisine and salsoline alkaloids, resulted in one-step and rather mild method for obtaining the corresponding bromomethyl derivatives of 1,3-thiazoline. The cyclization yield was affected by the base presence and the order in which the reagents were added. Molecular docking of the synthesized 1,3-thiazoline derivatives for putative antibacterial activity on the example of penicillin-binding target protein (PBP4) of bacteria *E. coli* “Homo sapiens” and *S. aureus* “Homo sapiens” showed that the compounds had insignificant values of binding energy at the level of selected comparators (*Cephalotin* and *Chloramphenicol*). The presence of natural alkaloids in the structure of thiazoline derivatives somewhat increased the affinity of those substrates for the target proteins selected.

## Figures and Tables

**Table 1:** Binding affinity (kcal/mol) of 1,3-thiazoline derivatives with active sites of penicillin binding proteins 4 (PBP4) *E. coli* “Homo sapiens” (PDB: 2EXB) and *S. Aureus* (PDB: 3HUN).

	Binding Energy (kcal/mol)	
**Ligand**	**Receptor**	
	2EXB	3HUN
*Cephalotin*	−7.0	-
*Chloramphenicol*	-	−6.8
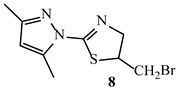	−5.2	−5.6
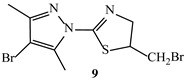	−5.2	−6.0
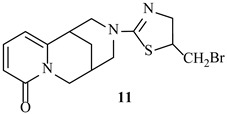	−6.6	−7.5
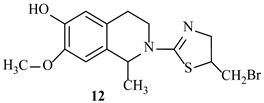	−6.3	−7.5
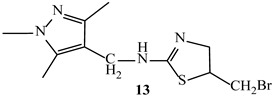	−5.6	−6.4

**Table 2:** Basic amino acid interactions and H-bonds.

Compound	Receptor	H-Bond	Residual Amino Acid Interactions	
			Pi-Alkyl, Alkyl, Pi-Sigma, Pi-Pi Stacked	Van-Der Walls Interactions
**8**	2EXB	SER306, ASN308	LEU421, PHE160, LEU359, ALA261	LYS305, LYS65, ARG361, SER357, GLY358
**9**		SER306	PHE160, LEU359, LEU421	LYS305, LYS65, ASN308, ALA61, ARG361
**11**		SER420	PHE160, LEU359, LEU421, ALA61	ASN308, LYS65, SER62, SER357, GLY358, ARG361
**12**		ASN308, PHE160	PHE160, ALA61, LEU421, LEU359	ARG361, GLY358, SER357, LYS65, SER306
**13**		SER420, SER306	PHE160, ALA61, LEU421, LEU359	ASN308, LYS417, LYS305, GLY358, ARG361
**8**	3HUN	SER116, SER75	ALA182, LEU115, PHE241	ARG186, ASN141, LYS78, SER262, SER139, LYS259, SER263, TYR291
**9**		SER116, SER75	PHE241, LEU115, ALA182	LYS259, SER139, ASN141, LYS78, ARG186
**11**		SER116, SER262, SER75	LEU115, PHE241	ALA182, GLU183, ARG186, GLU114, TYR291
**12**		ASP264, ARG186, GLU183, SER75	LEU115, PHE241, ALA182, TYR291	ASN141, LYS78, LYS259, SER116, SER262, SER139, SER263, GLU114
**13**		-	ALA74, ALA182, PHE241	ARG186, ASN72, THR180, GLY181, ASN141, SER75, LYS78, LYS259, SER139, SER116, LEU115

## Data Availability

Not applicable.

## Supplementary Materials

The following supporting information can be downloaded at: https://www.mdpi.com/article/10.3390/molecules27217598/s1, Pages 2–5, Experimental Procedures, Spectroscopic and physical data; Figures S1–S6, ^1^H and ^13^C NMR spectra; Figures S7–S11, Mass spectra; Tables S1–S3, X-Ray data; Table S4, Molecular docking data.
