# Supplementary material for: Synthesis, Structure and Molecular Docking of New 4,5-Dihydrothiazole Derivatives Based on 3,5-Dimethylpyrazole and Cytisine and Salsoline Alkaloids
Source: Molecules. 2022 Nov 5;27(21):7598. doi: 10.3390/molecules27217598 (PMC9655236; doi:10.3390/molecules27217598)
Supplement: Supplementary file 1 [file molecules-27-07598-s001.zip › molecules-1982642-supplementary.pdf]

Supporting information

Synthesis, Structure and Molecular Docking of New 4,5-Dihydrothiazole Derivatives Based on 3,5-Dimethylpyrazole and Cytisine and Salsoline Alkaloids

Marat K. Ibrayev [1,2,*], Oralgazy A. Nurkenov [1,3], Zhanar B. Rakhimberlinova [1], Altynaray T. Takibayeva [1], Irina V. Palamarchuk [4], Dastan M. Turdybekov [1], Assel A. Kelmyalene [1] and Ivan V. Kulakov [4]

[1] Department of Chemistry and Chemical Technology, Abylkas Saginov Karaganda Technical University, Ave. Nursultan Nazarbayev, 56, Karaganda 100027, Kazakhstan
[2] Faculty of Chemistry, Karaganda Buketov University, st. University 28, Karaganda 100024, Kazakhstan
[3] Institute of Organic Synthesis and Coal Chemistry of Republic of Kazakhstan, Alikhanova 1, Karaganda 100008, Kazakhstan
[4] Institute of Chemistry, Tyumen State University, 15a Perekopskaya St., Tyumen 625003, Russia
* Correspondence: mkibr@mail.ru; Tel.: +7-700-3106646

Table of contents

Experimental 1
General Information 1
Experimental Procedures 2
Spectroscopic and physical data 2
Copies of NMR Spectra of Products 6
Copies of MS Spectra of Products 13
X-Ray Structural Study of Product 16

X-Ray Structural Study of Product

Experimental

General Information

FTIR spectra were obtained with an Agilent Cary 630 spectrophotometer in a thin sample layer on a crystal attachment. ^{1}H and ^{13}C NMR spectra were recorded on a Bruker DRX400 (400 and 100 MHz, respectively) and Bruker AVANCE 500 (500 and 125 MHz, respectively) instruments using DMSO-d_6 the internal standard was TMS or residual solvent signals (2.49 and 39.9 ppm ^{1}H and for ^{13}C nuclei in DMSO-d_6).

Chromato-mass spectrometric studies were carried out on a Trace GC Ultra chromatograph with a DSQ II mass-selective detector in the electron ionization mode (70 eV) on a Thermo TR-5 MS quartz capillary column, 15 m long, 0.25 mm inner diameter, with a film thickness of the stationary phase of 0.25 µm. Splitless input mode was used. Carrier gas discharge 20 ml/min. The velocity of the carrier gas (helium) is 1 ml/min. Evaporator temperature 200°C, transition chamber temperature 200°C, ion source temperature 200°C. The temperature of the column thermostat was changed according to the program: from 15 (5 min delay) to 220°C at a rate of 20°C per minute, to 290° at a rate of 15° per minute. The total analysis time was 30 min. The volume of the injected sample is 1 µl. Chromatograms were recorded in TIC mode. The range of mass scanning is 30 - 450 amu.
Melting points were determined using a Stuart SMP10 hot bench. Monitoring of the reaction course and the purity of the products was carried out by TLC on Sorbfil plates and visualized using iodine vapor or UV light.

Experimental Procedures

N-allyl-4-bromo-3,5-dimethyl-1*H*-pyrazole-1-carbothioamide 5. A solution of 1.75 g (0.01 mol) of 4-bromo-3,5-dimethylpyrazole in 10 ml of 2-propanol was added dropwise to a solution of 1 g (0.01 mol) of allylisothiocyanate in 10 ml of 2-propanol at a temperature of 50°C. The solution was stirred for about 6 hours. After the solvent distillation and recrystallization of the residue from hexane, there was obtained 1.95 g (71%) of a white crystalline substance with m.p. 50-52 °C.

5-(bromomethyl)-2-(3,5-dimethyl-1*H*-pyrazol-1-yl)-4,5-dihydrothiazole 8 (General method). A solution of 0.96 g (10 mmol) of 3,5-dimethylpyrazole **1** in 7 ml of benzene was added within 30 minutes to a solution of 2.60 g (10 mmol) of 2,3-dibromopropylisothiocyanate **7** and 1.01 g (20 mmol) of triethylamine in 10 ml of abs. benzene with vigorous stirring and a temperature of 20 °C. The solution was heated for about 3 hours at a temperature of 40 °C. The precipitate of triethylamine hydrobromide was filtered off, washed with benzene. The benzene solution was evaporated to yield 2.63 g (96%) of a white crystalline substance with m.p. 101.5-102.5 °C (hexane-benzene).

2-(4-bromo-3,5-dimethyl-1*H*-pyrazol-1-yl)-5-(bromomethyl)-4,5-dihydrothiazole **9** was obtained by analogy with **8** from 2.60 g (10 mmol) of 2,3-dibromopropylisothiocyanate **7**, 1.01 g (20 mmol) of triethylamine and 1.75 g (10 mmol) of 4-bromo-3,5-dimethylpyrazole **2**. The yield was 2.29 g (65%); white crystals, m.p. was 106-107 °C (2-PrOH/ hexane, 2:1).

3-(5-(bromomethyl)-4,5-dihydrothiazol-2-yl)-1,2,3,4,5,6-hexahydro-8*H*-1,5-methanopyrido[1,2-a][1,5]diazocin-8-one **11** was obtained by analogy with **8** from 2.60 g (10 mmol) of 2,3-dibromopropylisothiocyanate **7**, 2.02 g (20 mmol) of triethylamine and 1.90 g (10 mmol) of cytisine **a**. The yield was 2.72 g (74%); white crystals, m.p. was 166-168°C (2-PrOH/hexane 1:1).

22-(5-(bromomethyl)-4,5-dihydrothiazol-2-yl)-7-methoxy-1-methyl-1,2,3,4-tetrahydroisoquinolin-6-ol **12** was obtained by analogy with **8** from 2.60 g (10 mmol) of 2,3-dibromopropylisothiocyanate **7**, 2.02 g (20 mmol) of triethylamine and 1.93 g (10 mmol) of salsoline **b**. The yield was 3.08 g (83%); white crystals, m.p. was 149-150°C (2-PrOH/hexane 1:1).

5-(bromomethyl)-N-((1,3,5-trimethyl-1*H*-pyrazol-4-yl)methyl)-4,5-dihydrothiazol-2-amine **13** was obtained by analogy with **8** from 2.60 g (10 mmol) of 2,3-dibromopropylisothiocyanate **7**, 2.02 g (20 mmol) of triethylamine and 1.39 g (10 mmol) of (1,3,5-trimethyl-1*H*-pyrazol-4-yl)methylamine **10**. The yield was 2.28 g (72%); white crystals, m.p. was 95-97 °C (PhH/hexane 1:1).

Spectroscopic and physical data

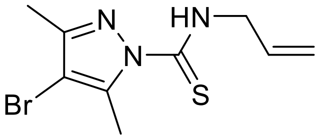<br>Chemical Formula: $C_9H_{12}BrN_3S$<br>Molecular Weight: 274,1800	**N-allyl-4-bromo-3,5-dimethyl-1*H*-pyrazole-1-carbothioamide 3.**<br>Yield: 1.94 g (71%); White crystals, mp. 50-52 °C (2-PrOH).<br>^{1}H NMR (500 МГц, DMSO-d$_6$, δ, ppm, *J*/ Hz): 2.08 (s, 3H, CH_3); 2.15 (s, 3H, CH_3), 4.22 - 4.37 (m, 2H, NH-C<u>H</u>$_2$-CH=), 5.17 (ddd, 1H, *J* = 16.72, 10.3, 1.5, =C-<u>H</u>$_a$), 5.29 (br. dd, 1H, *J* = 17.01, 10.2, =C-<u>H</u>$_b$), 5.85-5.97 (ddt, 1H, *J* = 17.62, 10.25, 5.10, -C<u>H</u>=CH$_a$H$_b$), 12.58 (br. s, 1H, N<u>H</u>-CH$_2$). ^{13}C NMR (101 MHz, DMSO-d$_6$) δ ppm 12.1, 12.7, 46.4, 98.0, 115.2, 135.1, 142.3, 148.2, 160.5.<br>MS (EI) *m/z* (I_{rel}, %): 275 $[M]^+$ (10), $[M]^+$ 273 (10), 242 (69), 240 (71), 176 (100), 175 (78), 174 (99), 95 (39), 42 (46), 41 (62), 39 (89).<br>Anal. calcd for $C_9H_{12}BrN_3S$: C, 39.78; H, 4.66; N, 15.81; found: C, 39.43; H, 4.41; N, 15.33.
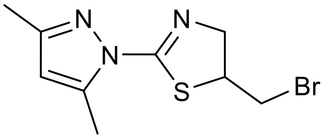<br>Chemical Formula: $C_9H_{12}BrN_3S$<br>Molecular Weight: 274,1800	**5-(bromomethyl)-2-(3,5-dimethyl-1*H*-pyrazol-1-yl)-4,5-dihydrothiazole 8**. Yield: 2.63 g (96%); White crystals, mp 101-102 °C (2-PrOH/ hexane, 2:1).<br>^{1}H NMR (500 МГц, DMSO-d$_6$, δ, ppm, *J*/ Hz): 2.15 (s, 3H, H_3); 2.47 (s, 3H, CH_3), 3.65 (dd, 1 H, BrCH$_{2a}$, *J* = 9.7, 9.7 Hz), 3.72 (dd, 1 H, BrCH$_{2b}$, *J* = 9.9, 9.8 Hz), 4.25 - 4.30 (m, 3H, =N-CH$_2$, S-CH), 6.15 (s, 1H, H-4 pyrazole).<br>^{13}C NMR (125 MHz, DMSO-d6) δ ppm 13.1, 13.5, 37.0, 50.1, 65.2, 110.0, 142.1, 150.3, 156.1.<br>Anal. calcd for $C_9H_{12}BrN_3S$: C, 39.81; H, 4.76; N, 15.52; found: C, 39.43; H, 4.41; N, 15.33.
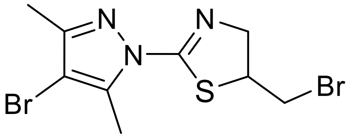<br>Chemical Formula: $C_9H_{11}Br_2N_3S$<br>Molecular Weight: 353,0760	**2-(4-bromo-3,5-dimethyl-1*H*-pyrazol-1-yl)-5-(bromomethyl)-4,5-dihydrothiazole 9.**<br>Yield: 2.29 g (65%); White crystals, mp 106-107 °C (2-PrOH/ hexane, 2:1).<br>^{1}H NMR (400 МГц, DMSO-d$_6$, δ, ppm, *J*/ Hz): 2.16 (s, 3H, H_3); 2.49 (s, 3H, CH_3), 3.64- 3.72 (m, 2 H, BrCH$_2$), 4.28 - 4.33 (m, 3H, =N-CH$_2$, S-CH).<br>^{13}C NMR (101 MHz, DMSO-d$_6$) δ ppm 12.1, 12.8, 37.0, 50.5, 65.2, 99.6, 139.9, 148.9, 156.<br>MS (EI) *m/z* (I_{rel}, %): 355 $[M]^+$ (5), $[M]^+$ 353 (10), 351 $[M]^+$ (5), 276 (5), 275 (12), 274 (95), 272 (100), 187 (15), 175 (78), 173 (90).<br>Anal. calcd for $C_9H_{11}Br_2N_3S$: C, 30.62; H, 3.14; N, 11.90; found: C, 30.94; H, 3.43; N,

	12.27.
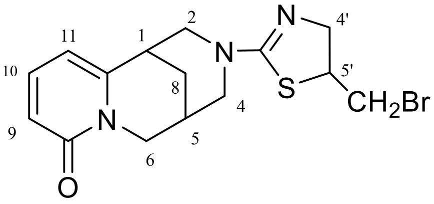<br>Chemical Formula: $C_{15}H_{18}BrN_3OS$<br>Molecular Weight: 368,29	3-(5-(bromomethyl)-4,5-dihydrothiazol-2-yl)-1,2,3,4,5,6-hexahydro-8*H*-1,5-methanopyrido[1,2-a][1,5]diazocin-8-one **11.** Yield: 2,72 g (74%); White crystals, mp 166-168°C (2-PrOH/hexane 1:1).<br>^{1}H NMR (400 MHz, DMSO-d_6, δ, ppm, *J*/ Hz): 1.97 (br. s, 2H, H-8), 2.56 (br. s, 1H, H-5), 3.16 (br. s, 1H, H-1), 3.24 (br. d, 2H, H-4_a), 3.4 (d, 1 H, *J*=5.0 Hz, H-2_a), 3.42 (dd, 2H, $BrCH_2$, *J*=9.8, 6.2 Hz), 3.45 (m, 1H, H-4_e) 3.70-3.80 (m, 2H, =N-CH_2), 3.82-3.86 (m, 1H, H-2_e), 3.98 (d, 1H, H-6_a, *J* =15.1 Hz), 4.02 (d, 1H, H-6_e, *J*= 9.6 Hz), 4.08-4.14 (m, 1H, S-CH), 6.23 (d, 1H, H-11, $J_{11,10}$ 6.9 Hz), 6.27 (d, 1H, H-9, $^3J_{9,10}$ = 8.7 Hz), 7.38 (dd, 1H, H-10, $^3J_{10,5}$ = 6.9 Гц, $J_{10,9}$ = 8.7 Hz).<br>^{13}C NMR (101 MHz, DMSO-d_6) δ ppm 25.0, 26.9, 33.9, 36.3, 48.6, 52.5, 54.1, 55.6 62.9, 104.9, 115.8, 138.9, 149.9, 160.9, 162.1.<br>MS (EI) *m/z* (I_{rel}, %): 288 (18), 287 (100), 160 (23), 146 (38), 141 (70), 113 (14), 100 (12).<br>Anal. calcd for $C_{15}H_{18}BrN_3OS$: C, 48.92; H, 4.93; N, 11.41; found: C, 49.18; H, 5.17; N, 11.27.
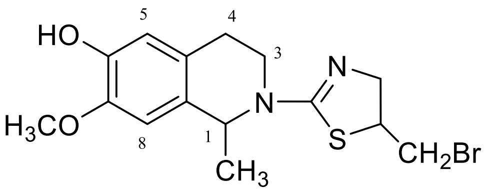<br>Chemical Formula: $C_{15}H_{19}BrN_2O_2S$<br>Molecular Weight: 371,29	(2-(5-(bromomethyl)-4,5-dihydrothiazol-2-yl)-7-methoxy-1-methyl-1,2,3,4-tetrahydro-isoquinolin-6-ol **12.** Yield: 3.08 g (83%); White crystals, mp 149-150°C (2-PrOH/hexane 1:1).<br>^{1}H NMR (400 MHz, DMSO-d_6, δ ppm): 1.37 (d, 3H, NCH-C<u>H</u>$_3$, *J* = 6.4); 2.54 (dt, 1H, H-4_{ax}, *J*=16.0, 3.2 Hz), 2.71 (ddd, 1 H, *J*=16.0, 10.8, 5.7 Hz, H-4_{eq}), 3.34-3.42 (m, 2H, H-3), 3.53 (t, 1 H, $BrCH_{2a}$, *J*=9.2 Hz), 3.61 (dd, 1 H, $BrCH_{2b}$, *J* = 10.1, 9.5 Hz), 3.72 (s, 3H, OCH_3), 3.85 - 3.96 (m, 2H, =N-CH_2), 4.16-4.22 (m, 1H, S-CH), 4.90 (q, 1 H, NC<u>H</u>-CH_3, *J*=6.3 Hz,), 6.50 (s, 1H, H-8), 6.70 (s, 1H, H-5), 8.77 (br. s, 1H, O-H).<br>^{13}C NMR (101 MHz, DMSO-d_6) δ ppm 21.6, 27.3, 36.5, 42.0, 52.3, 54.2, 55.8, 62.9, 110.5, 115.1, 125.4, 128.5, 145.0, 146.2, 159.5.<br>MS (EI) *m/z* (I_{rel}, %): 372 $[M]^+$ (10), $[M]^+$ 273 (10), 242 (69), 240 (71), 176 (100), 175 (78), 174 (99), 95 (39), 42 (46), 41 (62), 39 (89).<br>Anal. calcd for $C_{15}H_{19}BrN_2O_2S$: C, 48.52; H, 5.16; N, 7.54; found: C, 48.71; H, 5.31; N, 7.73.
	5-(bromomethyl)-N-((1,3,5-trimethyl-1*H*-pyra

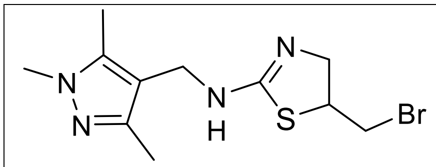<br>Chemical Formula: $C_{11}H_{17}BrN_4S$<br>Molecular Weight: 317,2490	zol-4-yl)methyl)-4,5-dihydrothiazol-2-amine<br>**13.** Yield: 2.28 g (72%) White crystals, mp 95-97 °C (PhH/hexane 1:1).<br>^{1}H NMR (500 MHz, DMSO-d$_6$, δ, ppm, *J*/ Hz): 2.06 (s, 3H, CH_3); 2.16 (s, 3H, CH_3), 3.60 (s, 3H, CH_3), 3.56-3.62 (m, 1 H, $BrCH_{2a}$), 3.66 (dd, 1 H, $BrCH_{2b}$, *J* = 9.9, 9.8 Hz), 3.87 - 3.93 (m, 2H, =N-CH_2), 4.08, 4.12 (2 d, 2 H, HN-CH_{2ab}, $^2J_{ab}$ = $^2J_{ba}$ = 14.3 Hz), 4.16-4.21 (m, 1H, S-CH), 7.37 (s, 1H, N-H).<br>MS (EI) *m/z* (I_{rel}, %): 318 $[M]^+$ (2), $[M]^+$ 316 (2), 351 $[M]^+$ (5), 138 (25), 123 (100), 122 (28), 56 (66), 55 (22), 41 (28), 39 (29).<br>Anal. calcd for $C_{11}H_{17}BrN_4S$: C, 41.65; H, 5.40; N, 17.66; found: C, 41.17; H, 5.74; N, 17.91.

Copies of NMR Spectra of Products

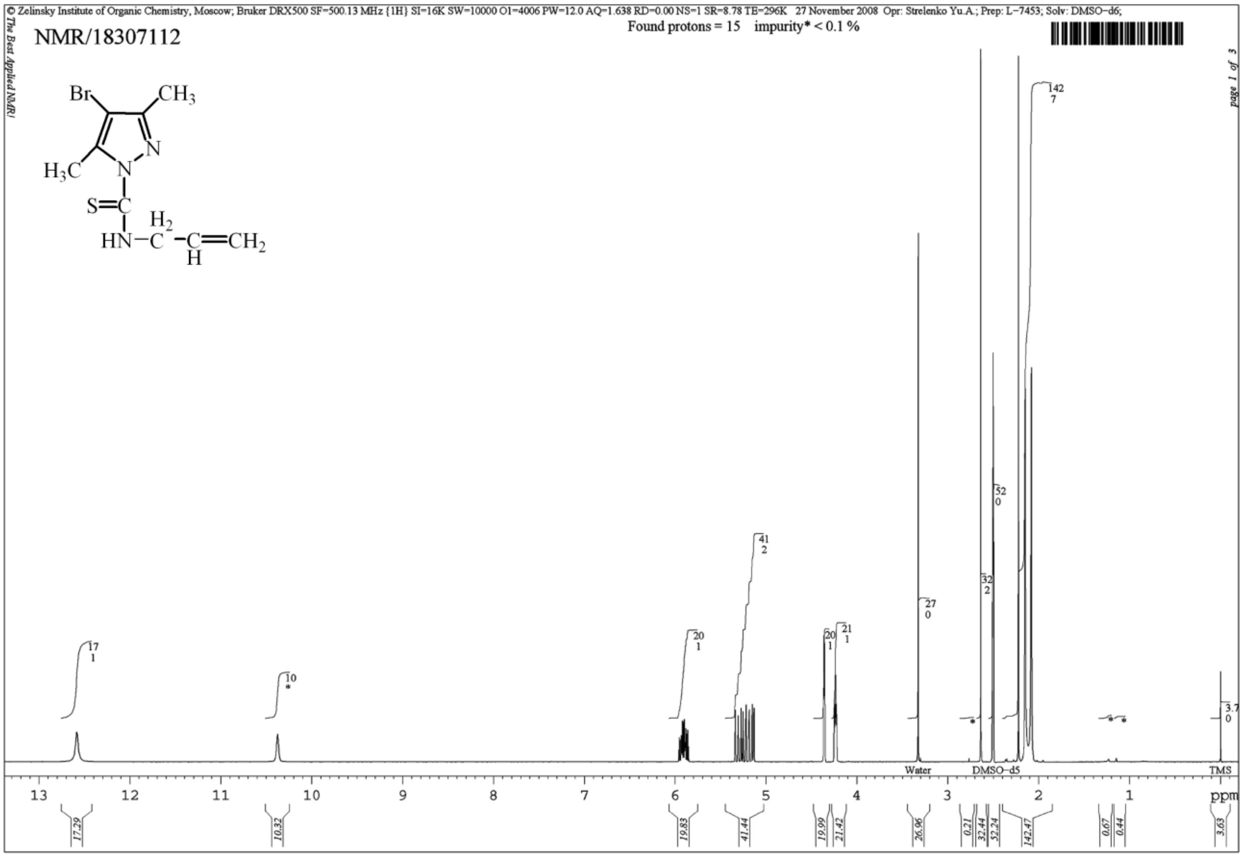

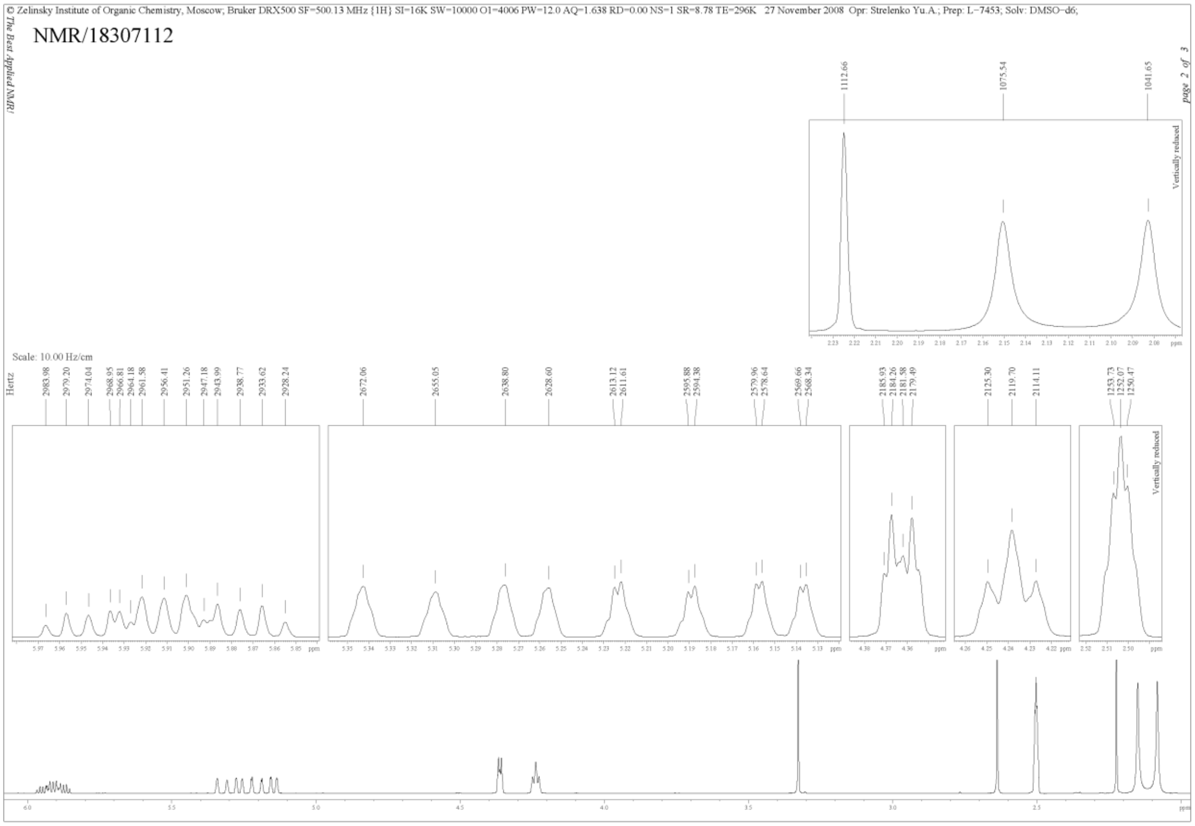

No.	(ppm)	(Hz)	Height
1	12.12	1219.1	-0.2817
2	12.68	1276.4	-0.3221
3	46.40	4669.3	0.1659
4	98.01	9861.9	0.2932
5	115.22	11594.0	0.2606

No.	(ppm)	(Hz)	Height
6	135.09	13593.1	-0.1314
7	142.34	14322.3	0.0826
8	148.24	14916.5	0.1430
9	160.46	16146.1	0.2636

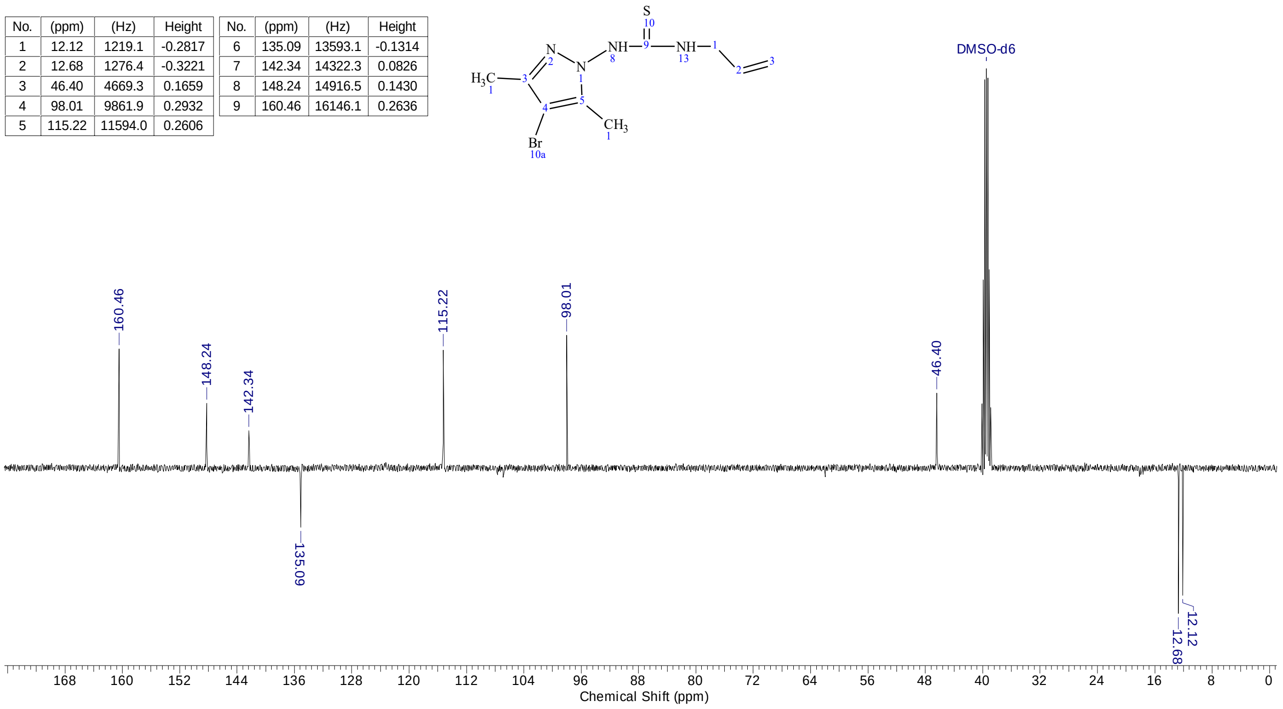

Figure S1. ^{1}H (500 MHz, DMSO-d6) and ^{13}C (100 MHz, DMSO-d6) NMR Spectra of **3**

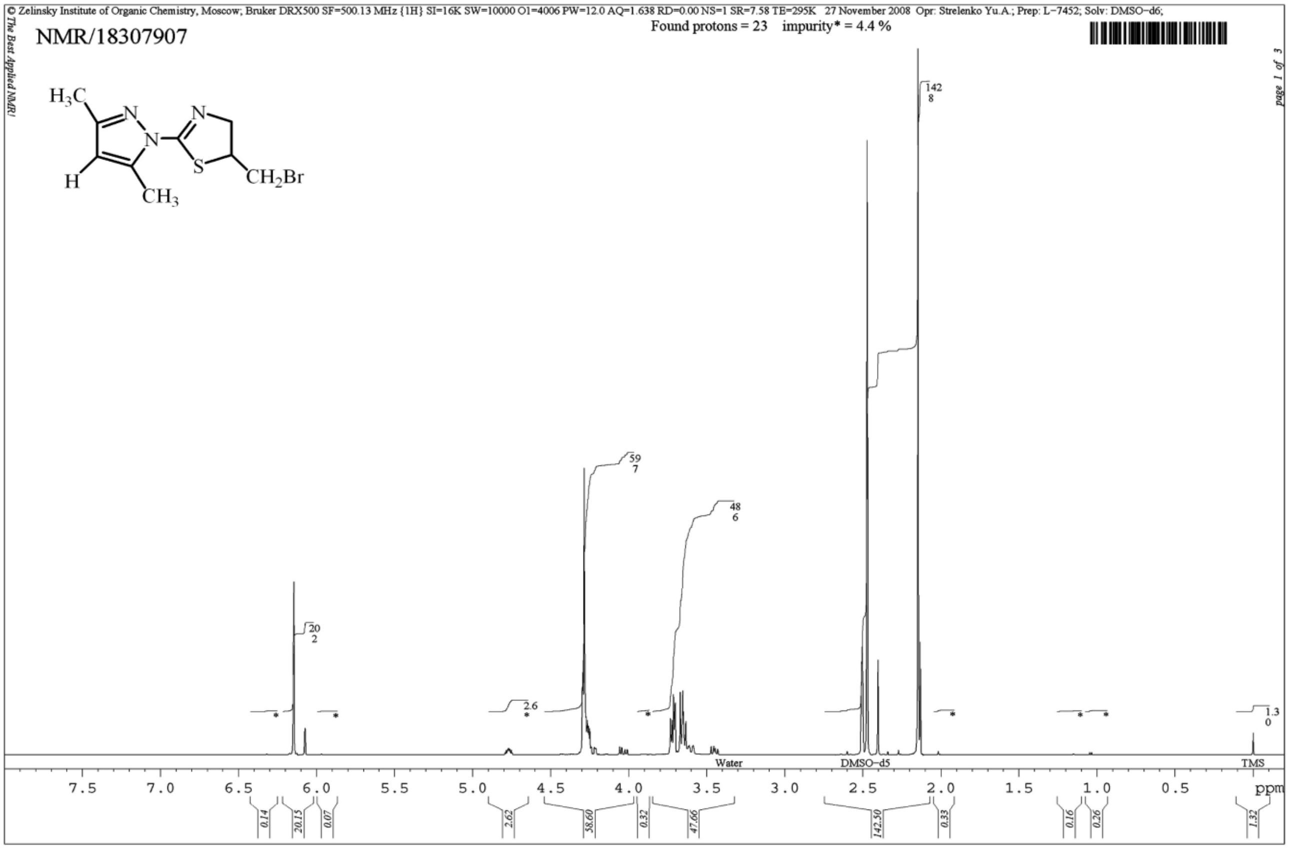

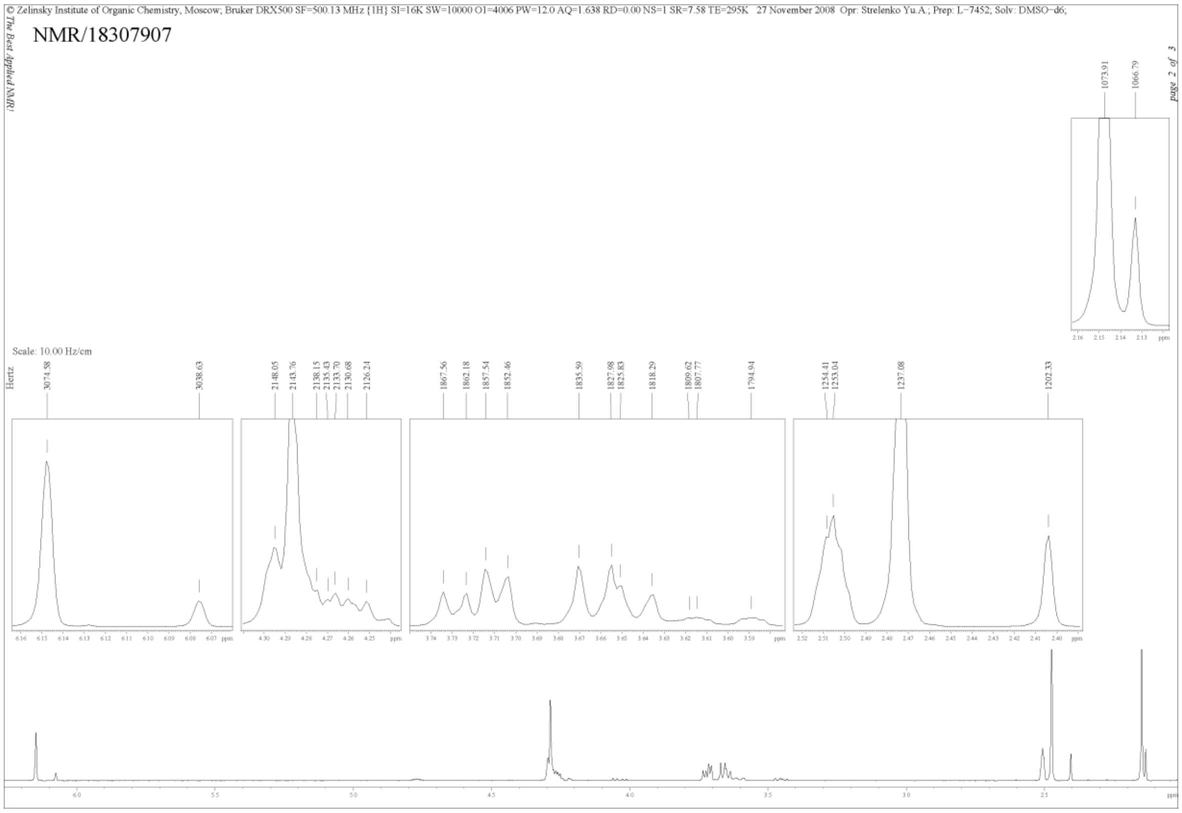

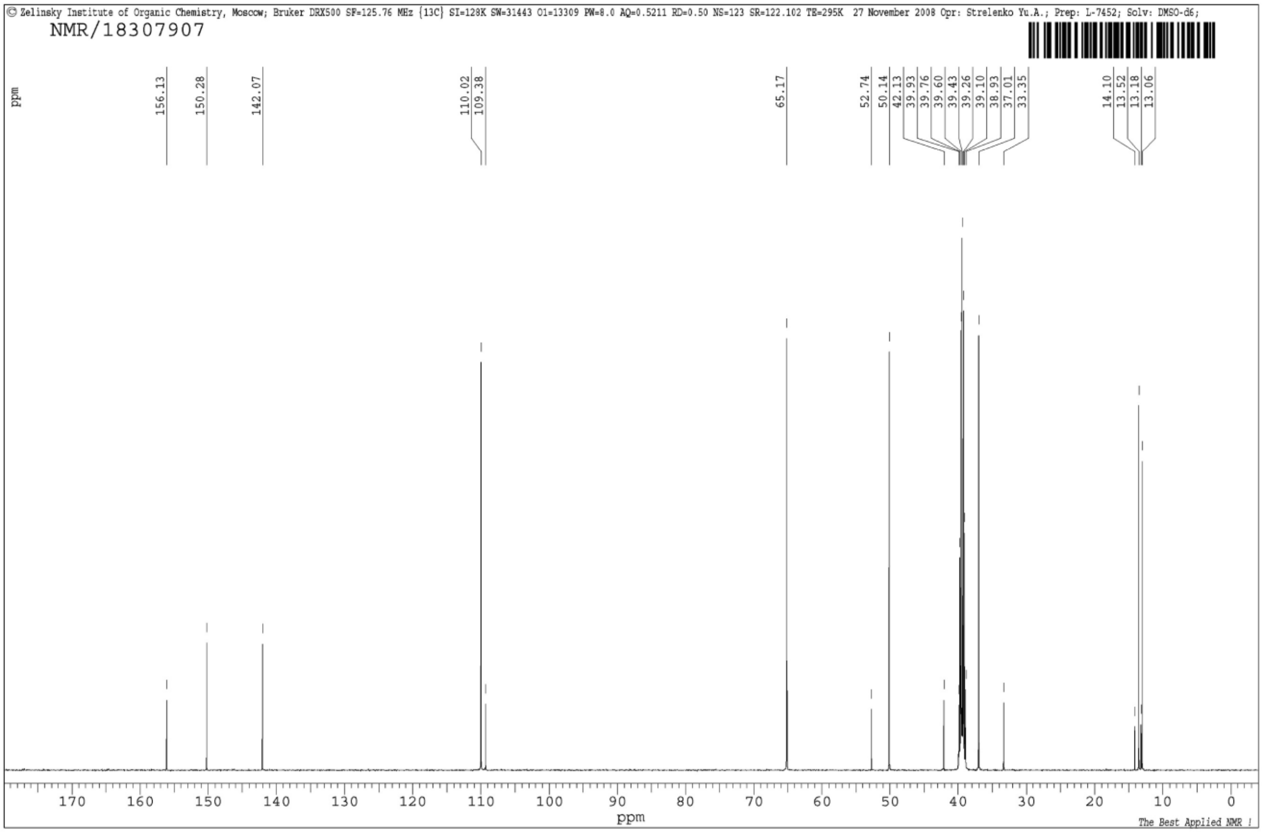

Figure S2. ^{1}H (500 MHz, DMSO-d6) and ^{13}C (125 MHz, DMSO-d6) NMR Spectra of **8**

No.	(ppm)	(Hz)	Height
1	2.16	862.2	0.2016
2	2.16	865.4	0.2049
3	2.49	997.3	0.3280
4	3.35	1339.4	1.0000
5	3.64	1453.4	0.0128
6	3.65	1459.8	0.0226

No.	(ppm)	(Hz)	Height
7	3.66	1463.0	0.0262
8	3.67	1467.2	0.0259
9	3.70	1479.5	0.0331
10	3.72	1489.1	0.0164
11	4.30	1719.0	0.1545

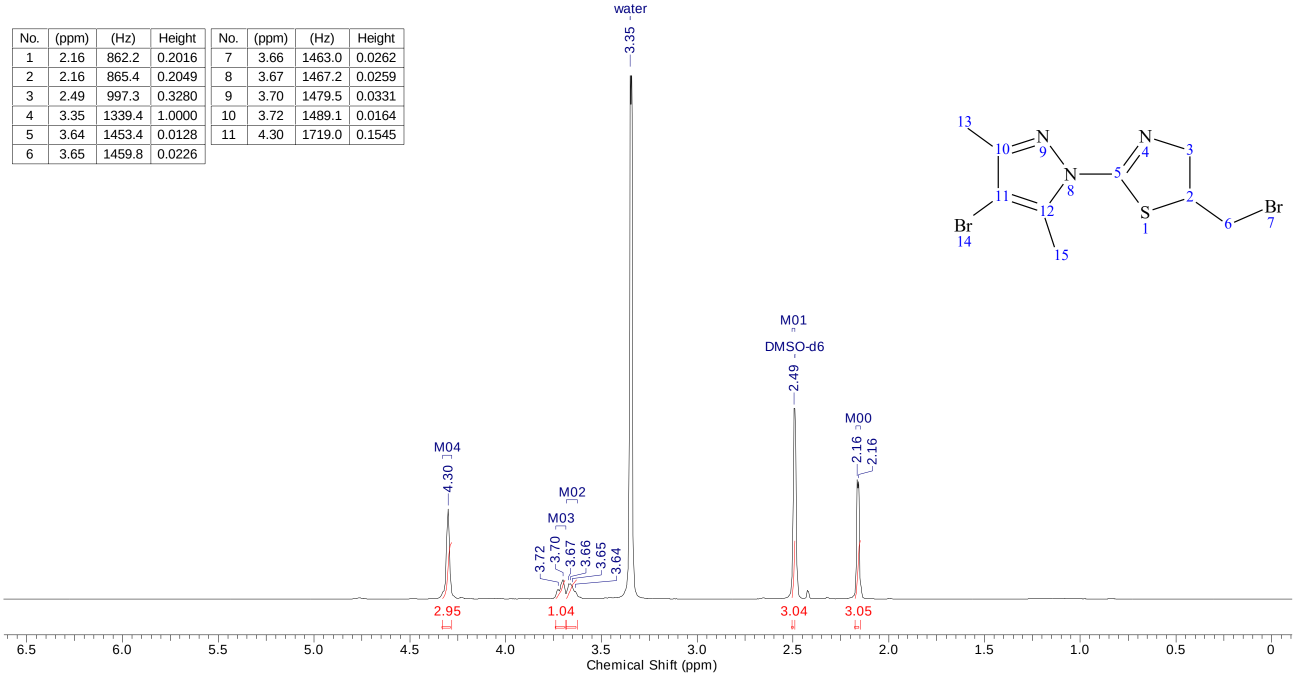

No.	(ppm)	(Hz)	Height
1	12.06	1212.3	0.0720
2	12.79	1286.1	0.0237
3	12.84	1290.9	0.0265
4	37.01	3720.6	0.0300
5	50.46	5073.0	0.0347

No.	(ppm)	(Hz)	Height
6	65.19	6552.9	0.0305
7	99.56	10008.1	0.0292
8	139.90	14063.4	0.0354
9	148.85	14963.4	0.0397
10	156.19	15701.4	0.0279

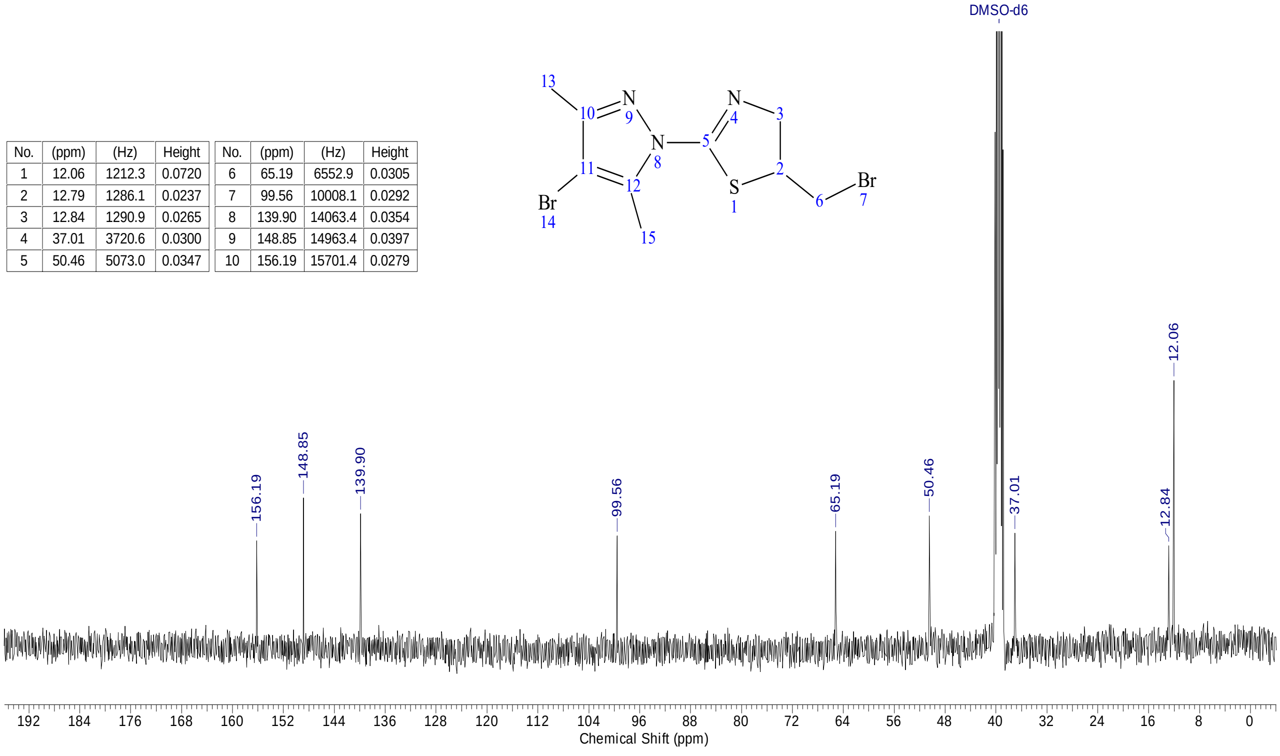

Figure S3. ^{1}H (400 MHz, DMSO-d6) and ^{13}C (100 MHz, DMSO-d6) NMR Spectra of **9**

No.	(ppm)	(Hz)	Height	No.	(ppm)	(Hz)	Height	No.	(ppm)	(Hz)	Height	No.	(ppm)	(Hz)	Height
1	1.97	787.1	1.0000	12	3.43	1370.1	0.5337	23	3.83	1530.8	0.4742	34	4.12	1645.7	0.2565
2	2.49	995.5	0.3361	13	3.44	1376.5	0.7091	24	3.86	1542.3	0.2249	35	4.13	1651.2	0.1141
3	2.56	1022.9	0.4489	14	3.45	1379.7	0.6537	25	3.86	1545.0	0.2037	36	4.14	1654.0	0.0854
4	3.16	1265.2	0.4731	15	3.70	1478.6	0.2303	26	3.97	1586.7	0.5301	37	6.22	2485.6	0.5295
5	3.23	1292.2	0.3295	16	3.71	1485.0	0.2732	27	4.00	1599.0	0.4236	38	6.23	2492.5	0.5508
6	3.26	1304.6	0.3492	17	3.73	1489.6	0.2994	28	4.01	1601.8	0.5847	39	6.26	2500.7	0.5506
7	3.31	1322.0	0.2679	18	3.74	1496.0	0.4059	29	4.03	1611.4	0.2840	40	6.28	2509.4	0.5326
8	3.35	1340.3	0.4731	19	3.76	1503.8	0.6302	30	4.08	1630.2	0.1375	41	7.36	2943.1	0.3383
9	3.37	1345.3	0.4475	20	3.78	1510.7	0.5731	31	4.09	1633.4	0.1684	42	7.38	2950.4	0.4117
10	3.40	1360.4	0.4749	21	3.80	1519.4	0.3433	32	4.10	1638.9	0.2910	43	7.40	2958.7	0.2894
11	3.42	1366.4	0.5791	22	3.82	1528.1	0.4906	33	4.11	1642.1	0.2412				

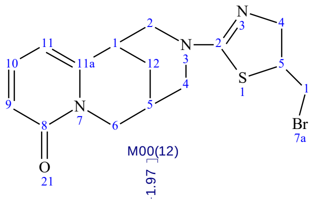

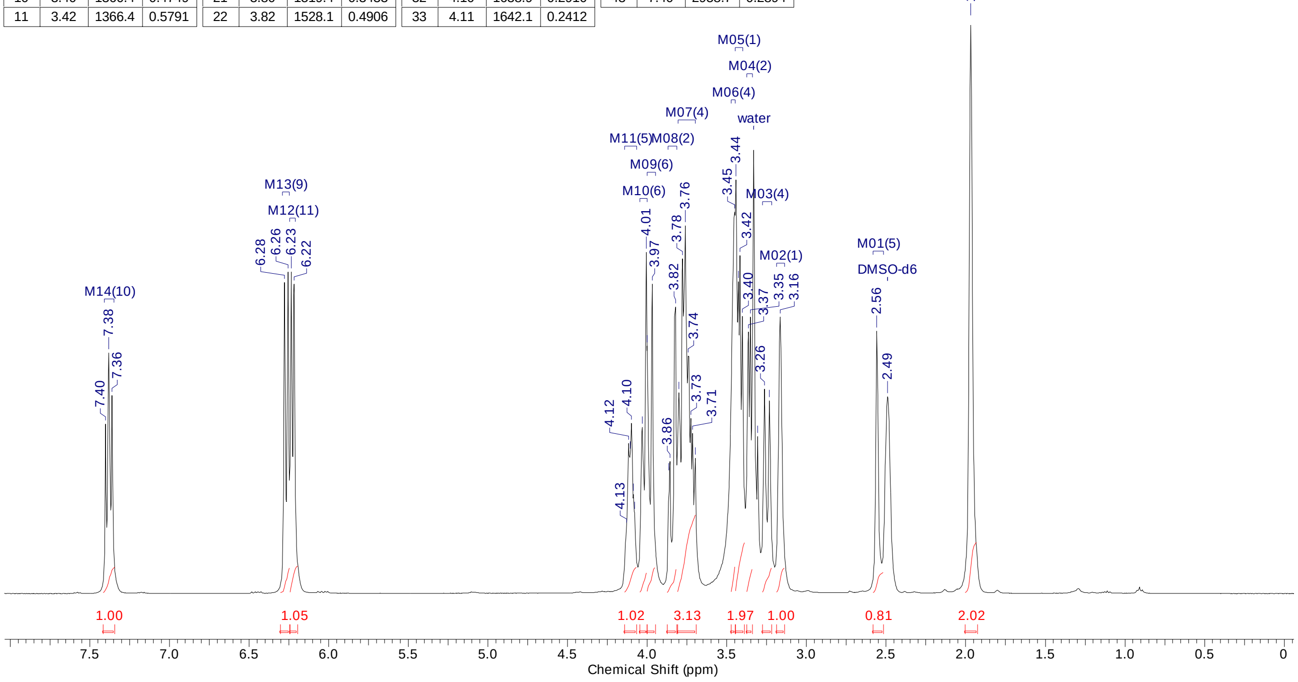

No.	(ppm)	(Hz)	Height	No.	(ppm)	(Hz)	Height	No.	(ppm)	(Hz)	Height
1	25.00	2512.9	0.0784	9	54.07	5435.3	0.0425	17	115.65	11626.0	0.0486
2	26.95	2709.4	0.0829	10	54.13	5441.0	0.0468	18	115.83	11644.2	0.0600
3	33.79	3396.6	0.0619	11	55.57	5585.8	0.0633	19	138.79	13952.2	0.0402
4	36.29	3647.7	0.0893	12	62.88	6320.9	0.0565	20	149.85	15064.0	0.1158
5	48.64	4889.9	0.0519	13	63.00	6333.4	0.0267	21	160.88	16172.0	0.0860
6	48.76	4901.4	0.0301	14	99.48	10000.5	0.0489	22	162.13	16298.5	0.1223
7	52.37	5264.7	0.0459	15	104.67	10521.9	0.0553				
8	52.50	5277.1	0.0631	16	104.85	10540.1	0.0591				

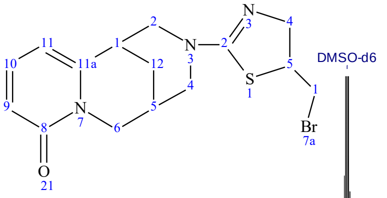

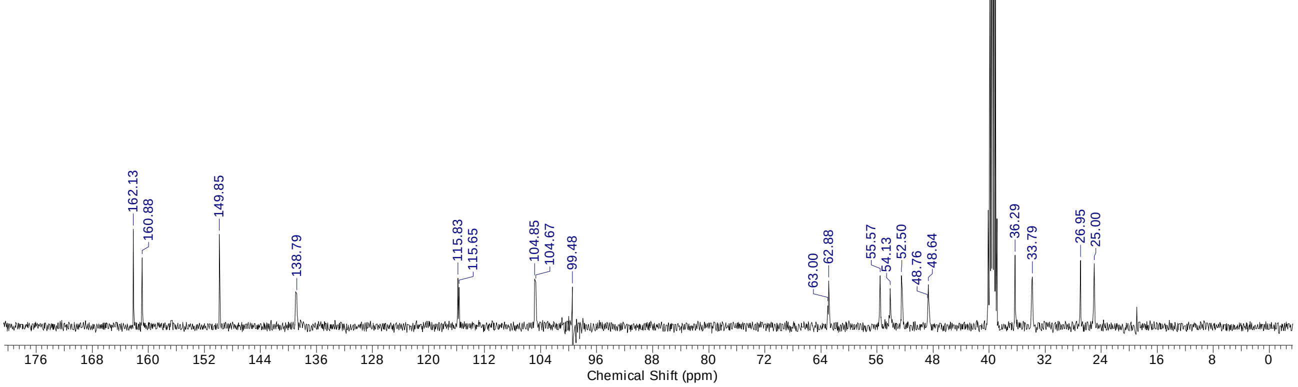

Figure S4. ^{1}H (400 MHz, DMSO-d6) and ^{13}C (100 MHz, DMSO-d6) NMR Spectra of **11**

No.	(ppm)	(Hz)	Height	No.	(ppm)	(Hz)	Height	No.	(ppm)	(Hz)	Height	No.	(ppm)	(Hz)	Height
1	1.37	546.7	0.3818	15	2.75	1099.9	0.0229	29	3.63	1451.1	0.0580	43	4.19	1674.6	0.0500
2	1.38	553.1	0.4115	16	3.34	1337.1	0.0456	30	3.72	1487.3	1.0000	44	4.20	1677.8	0.0519
3	2.52	1006.0	0.0292	17	3.35	1341.2	0.0517	31	3.85	1540.4	0.0333	45	4.20	1680.1	0.0416
4	2.52	1009.2	0.0460	18	3.38	1350.4	0.0724	32	3.87	1547.7	0.0345	46	4.21	1683.8	0.0259
5	2.53	1012.4	0.0315	19	3.38	1352.2	0.0707	33	3.89	1554.6	0.0987	47	4.22	1687.0	0.0186
6	2.56	1022.0	0.0467	20	3.39	1354.0	0.0672	34	3.91	1561.5	0.1252	48	4.88	1950.3	0.0227
7	2.56	1025.2	0.0718	21	3.41	1361.4	0.0603	35	3.92	1566.5	0.1301	49	4.89	1956.7	0.0630
8	2.57	1028.4	0.0456	22	3.42	1365.5	0.0496	36	3.93	1569.7	0.1253	50	4.91	1963.1	0.0645
9	2.67	1067.4	0.0298	23	3.50	1401.2	0.0593	37	3.95	1580.7	0.0379	51	4.93	1969.1	0.0247
10	2.68	1073.3	0.0372	24	3.53	1410.8	0.1223	38	3.96	1583.9	0.0349	52	6.50	2597.4	0.2730
11	2.70	1078.3	0.0376	25	3.55	1419.5	0.0995	39	4.16	1662.2	0.0210	53	6.70	2679.3	0.2812
12	2.71	1083.8	0.0525	26	3.59	1435.5	0.0905	40	4.17	1665.4	0.0278	54	8.77	3506.8	0.0223
13	2.72	1088.9	0.0272	27	3.61	1441.5	0.1052	41	4.18	1669.1	0.0420				
14	2.74	1094.4	0.0261	28	3.62	1445.6	0.0621	42	4.18	1671.4	0.0558				

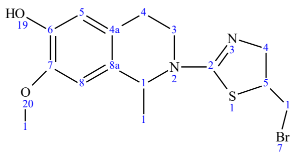

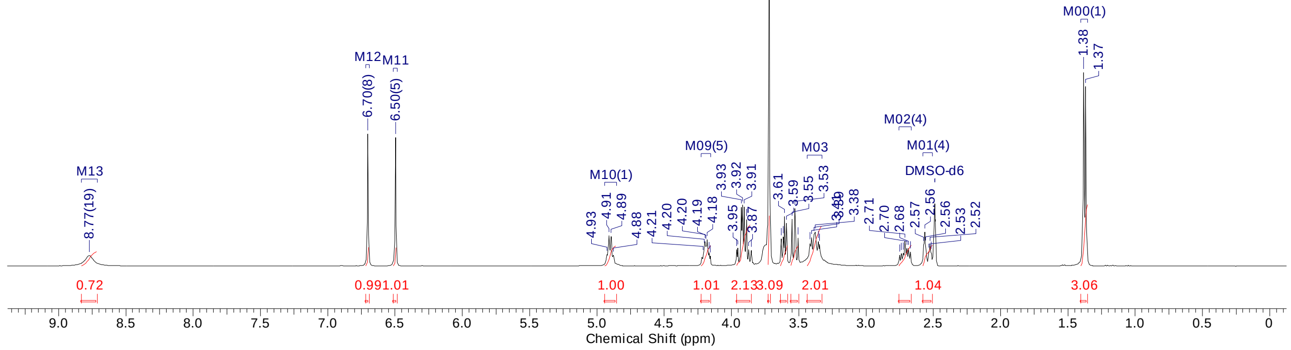

No.	(ppm)	(Hz)	Height	No.	(ppm)	(Hz)	Height	No.	(ppm)	(Hz)	Height
1	21.49	2160.2	0.0378	8	53.96	5424.7	0.0257	15	115.02	11562.8	0.0688
2	21.59	2170.7	0.0389	9	54.16	5444.9	0.0305	16	115.11	11571.4	0.0576
3	27.34	2748.7	0.0473	10	55.62	5591.5	0.0455	17	125.43	12608.5	0.0970
4	36.49	3667.9	0.0471	11	55.77	5605.9	0.0476	18	128.53	12920.9	0.0769
5	41.96	4218.0	0.0264	12	62.89	6321.9	0.0283	19	145.02	14578.1	0.1117
6	52.17	5244.6	0.0388	13	110.49	11107.5	0.0610	20	146.20	14697.0	0.1065
7	52.31	5258.9	0.0358	14	110.53	11111.3	0.0656	21	159.52	16035.9	0.0386

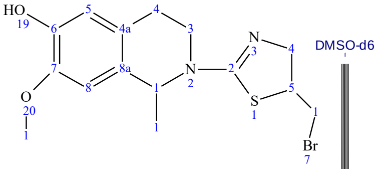

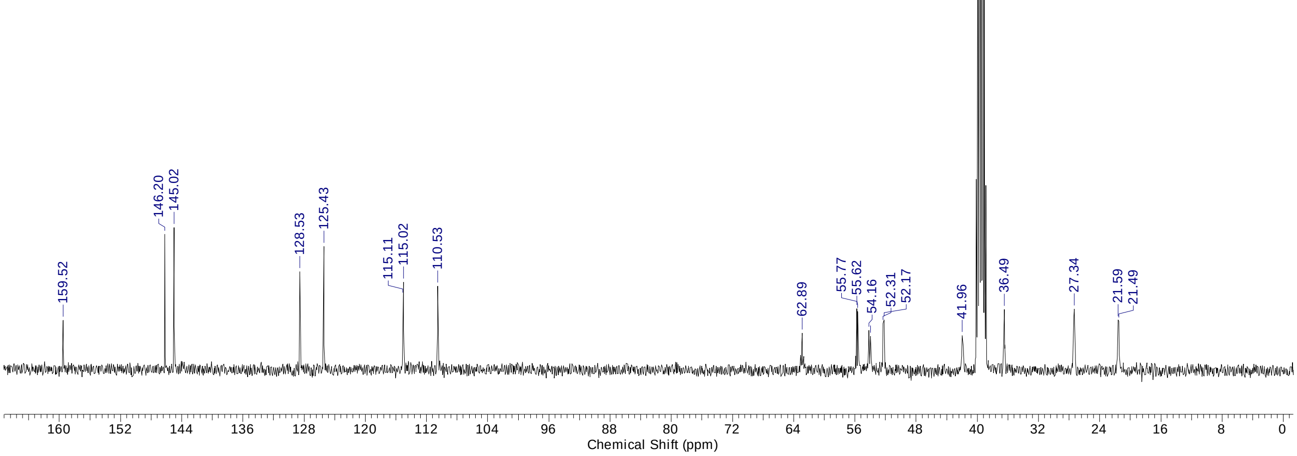

Figure S5. ^{1}H (400 MHz, DMSO-d$_6$) and ^{13}C (101 MHz, DMSO-d$_6$) NMR Spectra of **12**

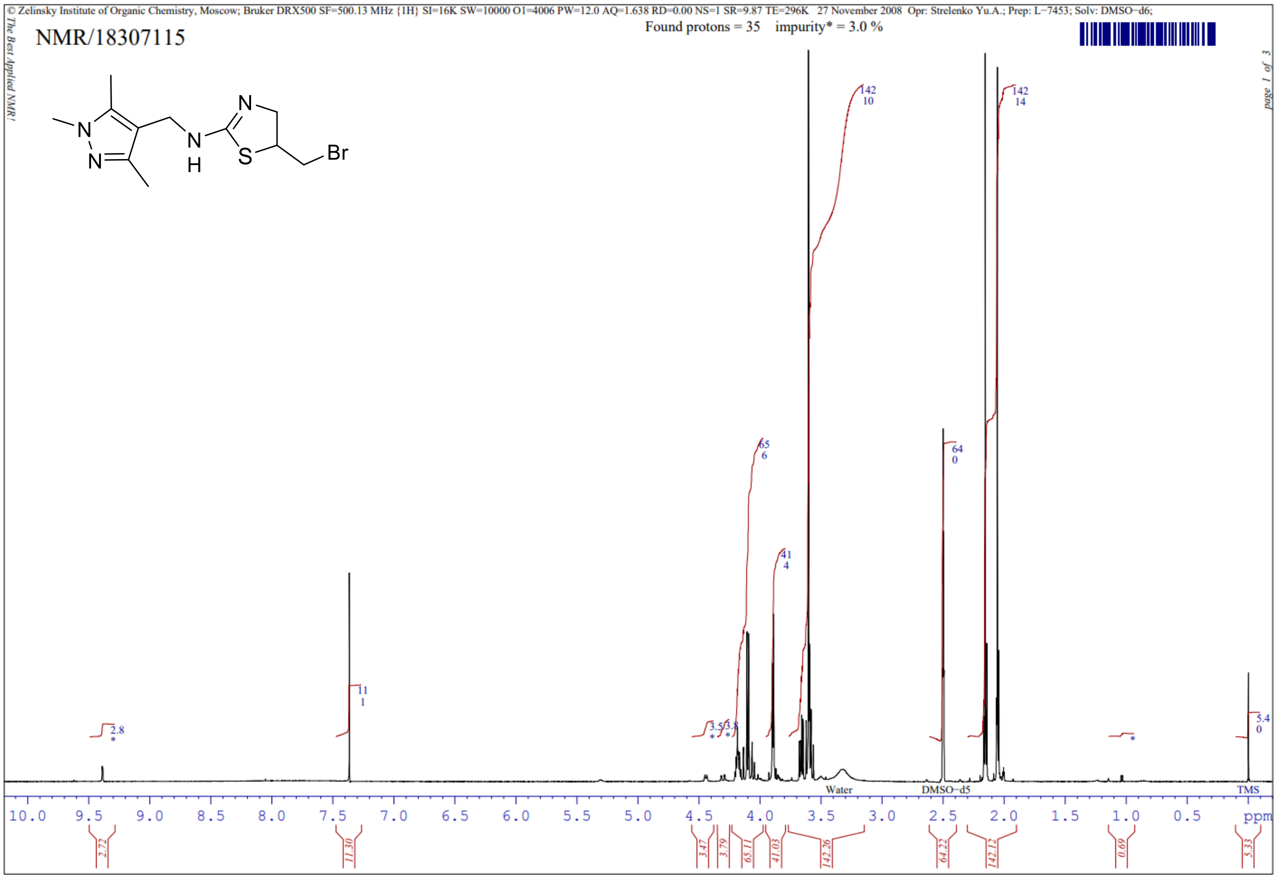

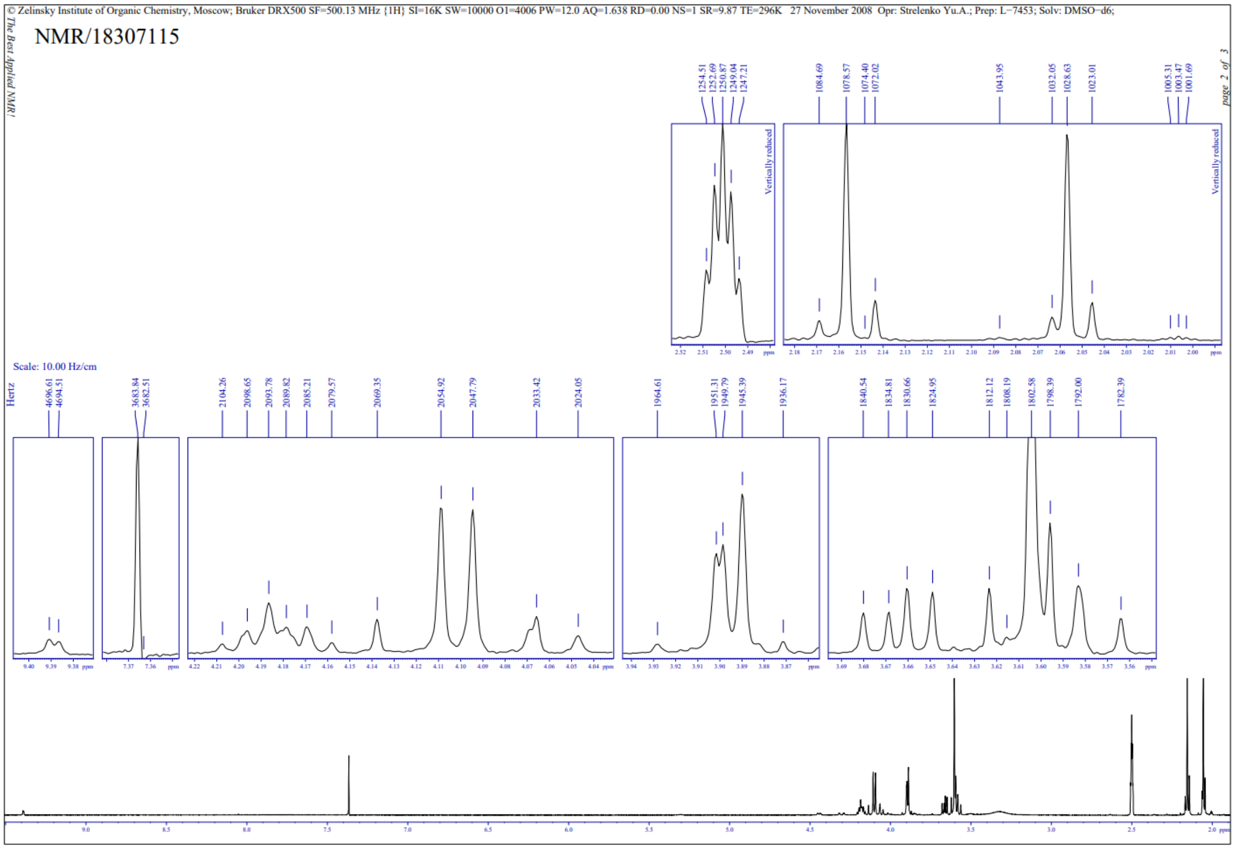

Figure S6. ^{1}H (500 MHz, DMSO-d_6) NMR Spectra of **13**

Copies of MS Spectra of Products

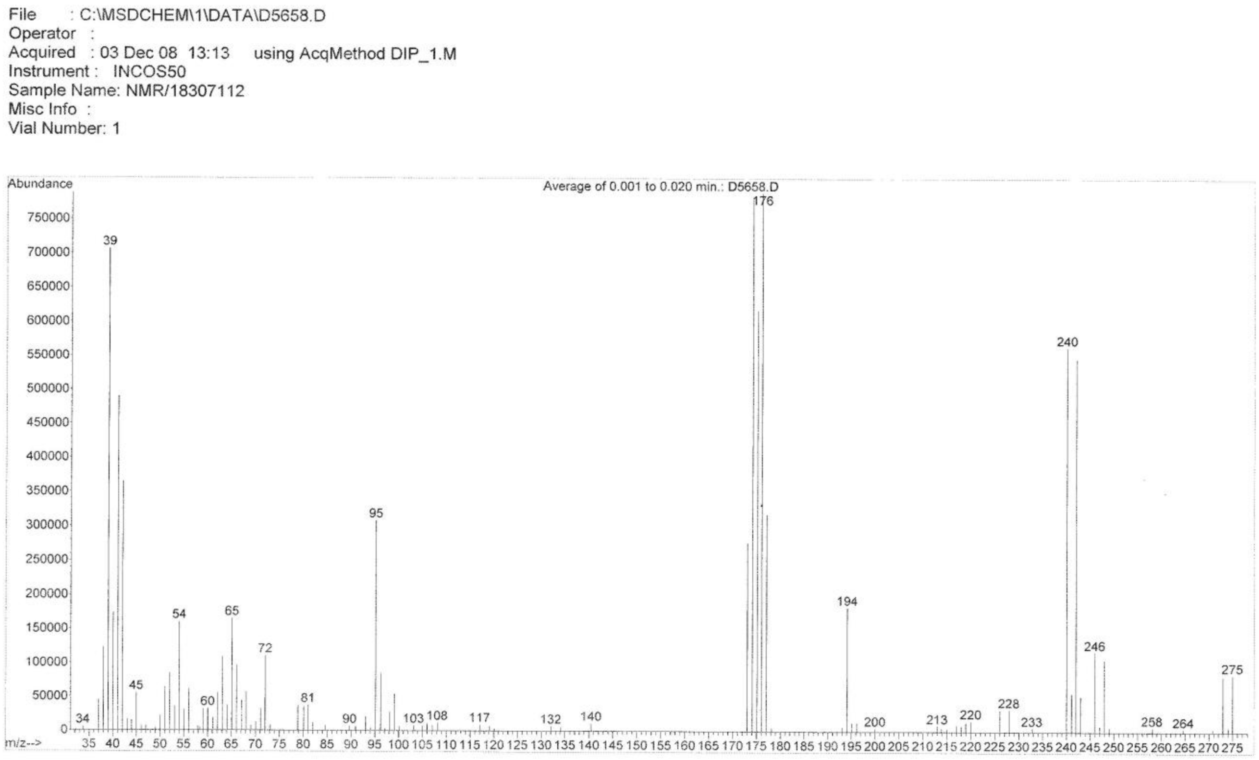

Figure S7. Mass spectrum of **3**

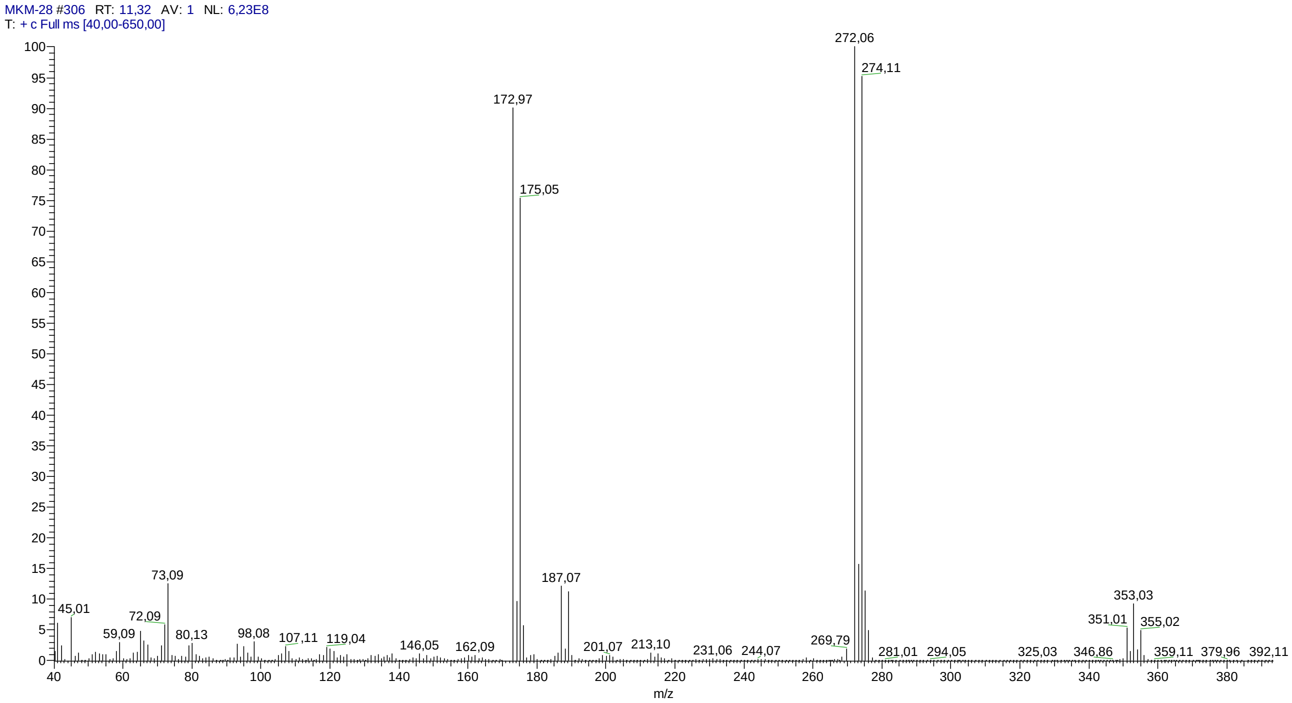

Figure S8. Mass spectrum of **9**

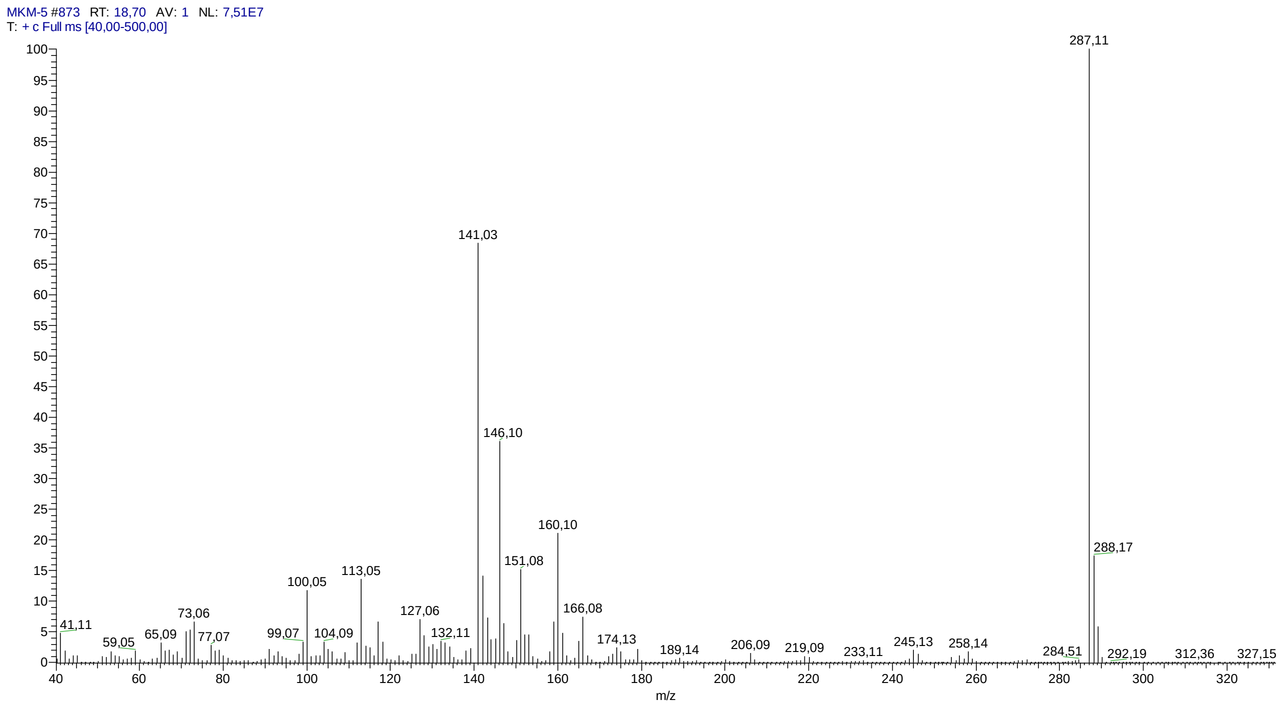

Figure S9. Mass spectrum of **11**

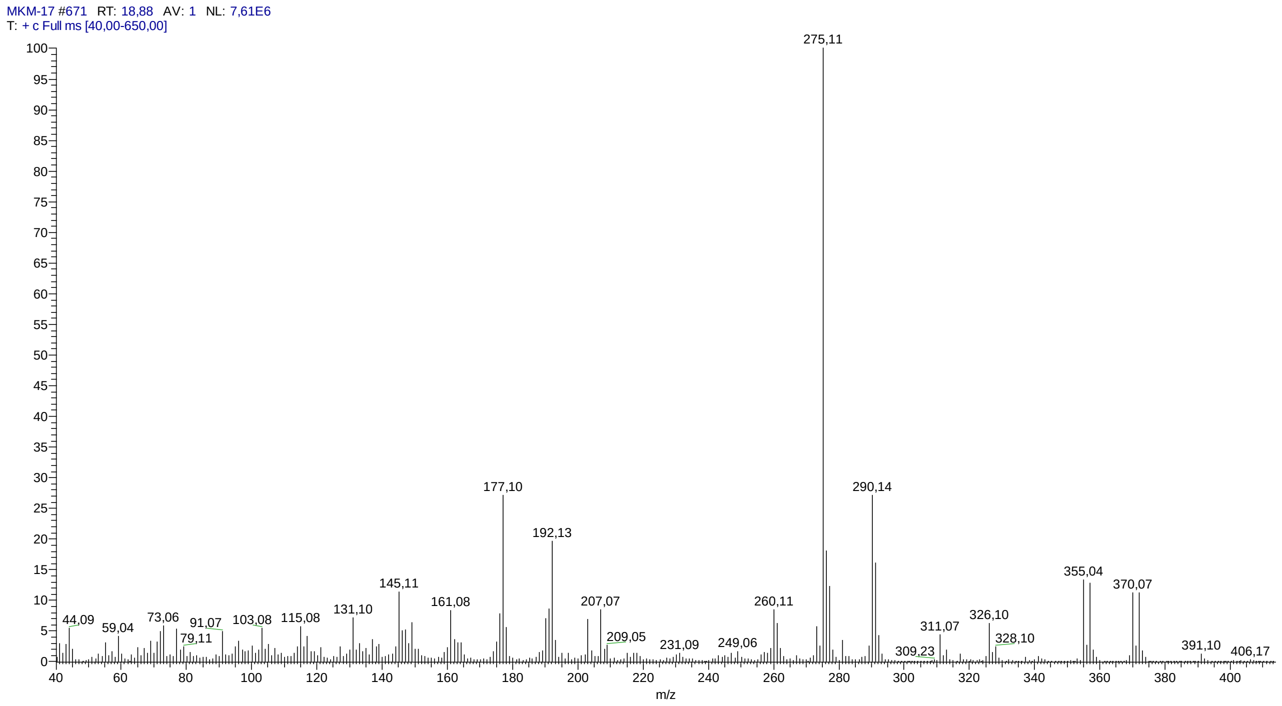

Figure S10. Mass spectrum of **12**

```
Mass spectrum  NMR/18307115: formula C11H17BrN4S, mol. mass 317.25
Max intensity: 999 for mass 123
Discrimination level for relative intensity: 5.0%,
whithin the interval of (molecular mass+50): 0.5% (marked with -->)
One symbol '*' on graphic = 5% of maximum relative intensity

 Mass  Intens.  Rel.Int(%)     |   Mass    *** Graphic ***    Rel.Int(%)
 39      292    29.23          |    39  *****                   29.23
 40       85     8.51          |    40  *                        8.51
 41      279    27.93          |    41  *****                   27.93
 42      234    23.42          |    42  ****                    23.42
 43       51     5.11          |    43  *                        5.11
 45      165    16.52          |    45  ***                     16.52
 53       93     9.31          |    53  *                        9.31
 54       72     7.21          |    54  *                        7.21
 55      216    21.62          |    55  ****                    21.62
 56      656    65.67          |    56  *************           65.67
 58       50     5.01          |    58  *                        5.01
 59      118    11.81          |    59  **                      11.81
 66      101    10.11          |    66  **                      10.11
 73       64     6.41          |    73  *                        6.41
 81       70     7.01          |    81  *                        7.01
122      284    28.43          |   122  *****                   28.43
123      999   100.00          |   123  ********************   100.00
124       92     9.21          |   124  *                        9.21
138      246    24.62          |   138  *****                   24.62
155       82     8.21          |   155  *                        8.21
237       82     8.21          |   237  *                        8.21
316       19     1.90         -->  316                           1.90
317        1     0.10  mol.mass:   317                           0.10
318       19     1.90         -->  318                           1.90
```

Figure S11. Mass spectrum of **13**

X-Ray Structural Study of Product

Cell parameters and intensities of 1480 independent reflections were measured on a Bruker APEX-II CCD diffractometer, MoKα-radiation, graphite monochromator, θ/2θ-scan, 2θ≤60°. There were rhombic crystals, a=5.5149(5), b=18.4636(7), c=20.7277(11) Å, V= 2110.6(2)Å3, d_{calc}= 1.600g/cm^3, Z=8 ($C_9H_{13}N_3SBr$), space group Pbca. The structure was elucidated by the direct method and refined by full-matrix least squares in the anisotropic approximation for non-hydrogen atoms. The H atoms were calculated geometrically and planted according to the "rider" type. The calculations used 958 reflections with I>2σ(I). Final divergence factors were R=0.082 and WR2= 0.227. The structure was solved and refined using the programs "SHELXS-97" and "SHELXL-97". The structure geometrical parameters were deposited with the CSDC (CCDC 723563).

Table S1. Bond lengths (d, Å) in molecule 8

Bond	d	Bond	d
S_1C_7	1.744(3)	O_2C_{10}	1.218(4)
S_1C_8	1.764(5)	O_3C_{13}	1.442(5)
C_1C_2	1.374(4)	N_3C_7	1.267(4)
C_1C_5	1.376(5)	N_4C_{10}	1.359(5)
C_1C_6	1.503(4)	N_4C_7	1.391(4)
N_1C_3	1.299(5)	C_4C_5	1.376(4)
N_1C_4	1.330(4)	C_8C_9	1.426(7)
O_1C_6	1.222(4)	C_9C_{11}	1.497(7)
N_2C_6	1.340(4)	C_9C_{10}	1.515(6)
N_2N_3	1.414(3)	$C_{12}C_{13}$	1.429(8)
C_2C_3	1.379(5)	$C_{13}C_{14}$	1.465(7)

Table S2. Bond angles (, deg) in molecule 8

Angle	degrees	Angle	degrees
$C_7S_1C_8$	100.82(18)	$N_2C_6C_1$	115.8(3)
$C_2C_1C_5$	116.6(3)	$N_3C_7N_4$	116.3(3)
$C_2C_1C_6$	118.2(3)	$N_3C_7S_1$	123.3(2)
$C_5C_1C_6$	125.3(3)	$N_4C_7S_1$	120.4(2)
$C_3N_1C_4$	117.0(3)	$C_9C_8S_1$	118.5(4)
$C_6N_2N_3$	120.0(3)	$C_8C_9C_{11}$	120.4(5)
$C_1C_2C_3$	119.7(3)	$C_8C_9C_{10}$	112.3(5)
$C_7N_3N_2$	112.7(3)	$C_{11}C_9C_{10}$	112.8(4)
$N_1C_3C_2$	123.7(3)	$O_2C_{10}N_4$	118.8(3)
$C_{10}N_4C_7$	128.3(3)	$O_2C_{10}C_9$	123.1(4)
$N_1C_4C_5$	123.3(3)	$N_4C_{10}C_9$	118.1(3)
$C_1C_5C_4$	119.5(3)	$C_{12}C_{13}O_3$	112.2(4)
$O_1C_6N_2$	123.1(3)	$C_{12}C_{13}C_{14}$	118.5(6)
$O_1C_6C_1$	121.0(3)	$O_3C_{13}C_{14}$	109.0(4)

Table S3. Torsion angles (, deg) in molecule 8

Angle	degrees	Angle	degrees
C5C1C2C3	-4.7(6)	N2N3C7N4	-177.4(3)
C6C1C2C3	174.2(4)	N2N3C7S1	-0.1(4)
C6N2N3C7	-90.0(4)	C10N4C7N3	-166.1(3)
C4N1C3C2	2.3(7)	C10N4C7S1	16.5(5)
C1C2C3N1	1.7(8)	C8S1C7N3	-177.9(4)
C3N1C4C5	-3.4(7)	C8S1C7N4	-0.7(4)
C2C1C5C4	3.7(6)	C7S1C8C9	-35.0(6)
C6C1C5C4	-175.1(4)	S1C8C9C11	-167.4(5)
N1C4C5C1	0.3(7)	S1C8C9C10	56.1(8)
N3N2C6O1	5.2(5)	C7N4C10O2	-179.8(4)
N3N2C6C1	-178.2(3)	C7N4C10C9	2.5(6)
C2C1C6O1	13.8(5)	C8C9C10O2	143.0(5)
C5C1C6O1	-167.5(4)	C11C9C10O2	3.1(8)
C2C1C6N2	-162.8(4)	C8C9C10N4	-39.4(8)
C5C1C6N2	15.9(5)	C11C9C10N4	-179.3(5)

Table S4. Complexes between synthesized derivatives **8, 9, 11-13** and active sites of proteins (PDB: 2EXB, 3HUN)

<table>
<tr><th colspan="2">2EXB</th><th colspan="2">3HUN</th></tr>
<tr>
<td>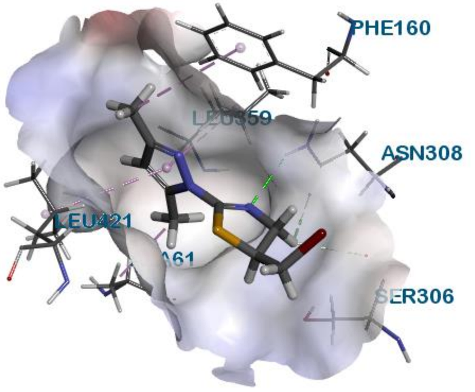

8</td>
<td>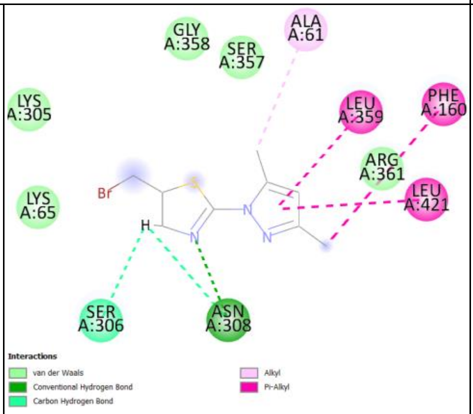

8</td>
<td>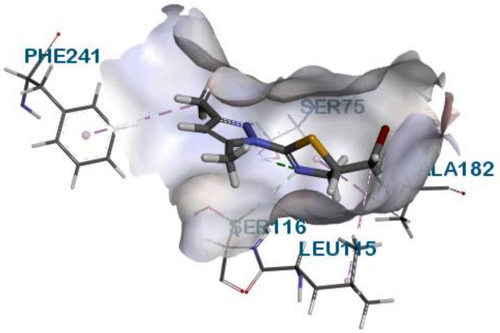

8</td>
<td>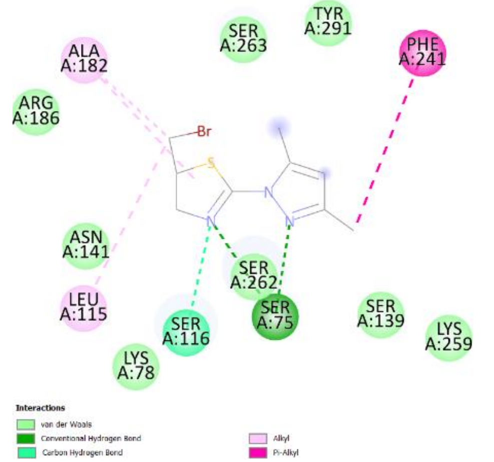

8</td>
</tr>
<tr>
<td>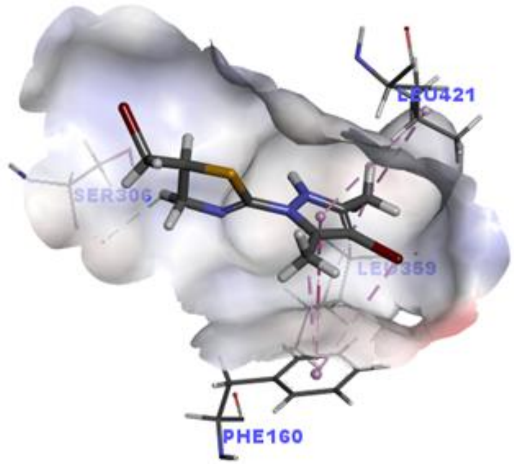

9</td>
<td>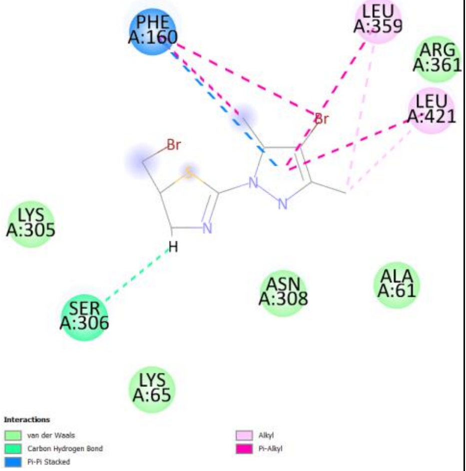

9</td>
<td>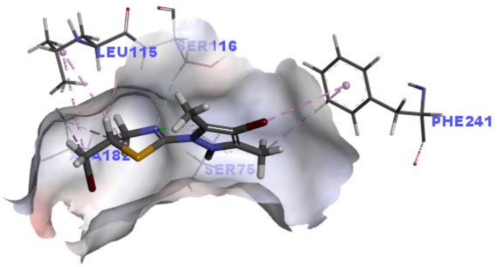

9</td>
<td>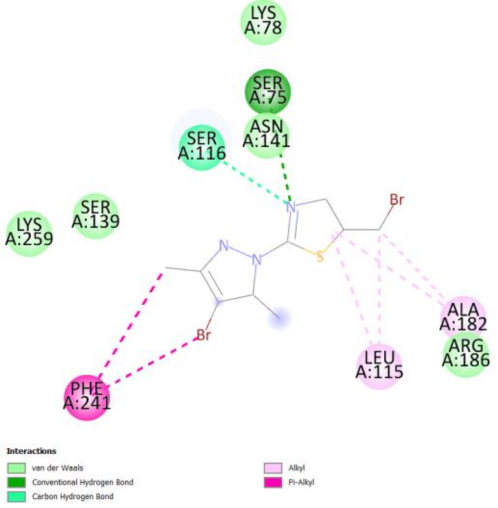

9</td>
</tr>
</table>

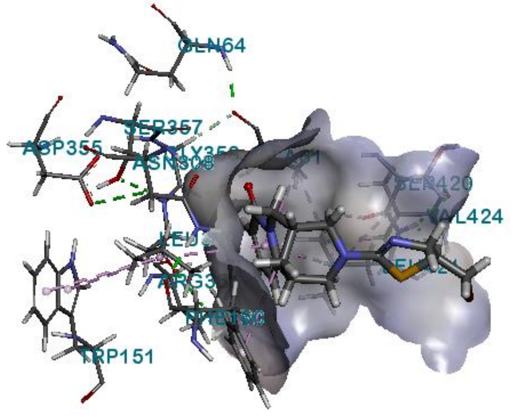

11

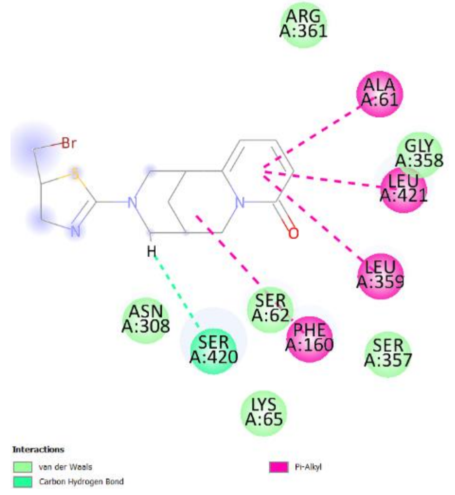

11

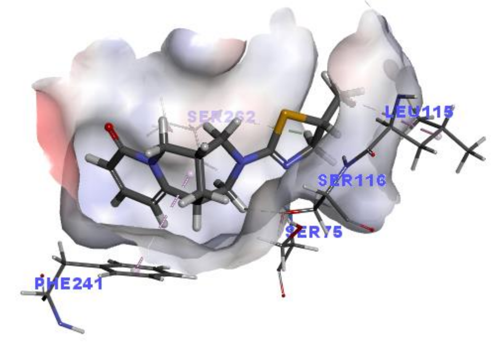

11

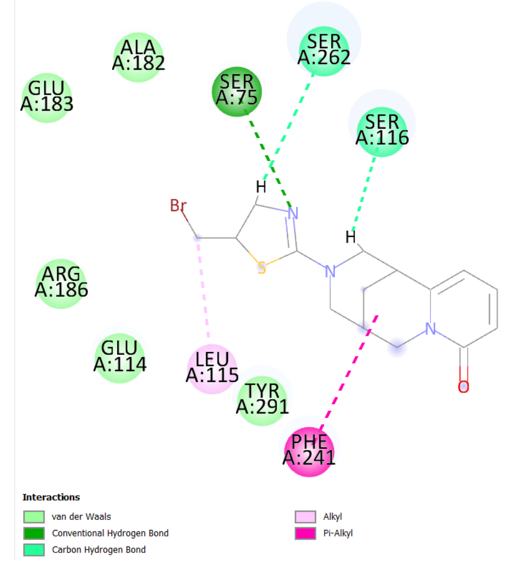

11

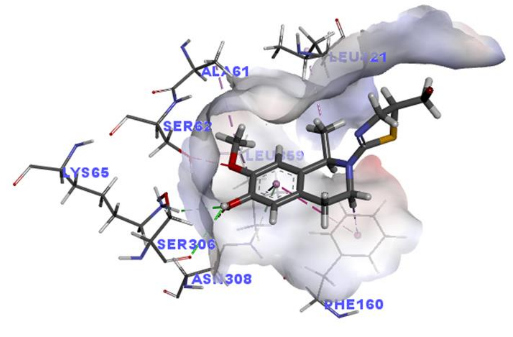

12

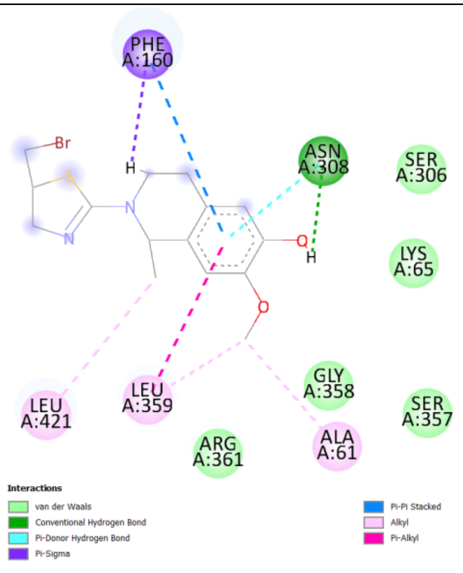

12

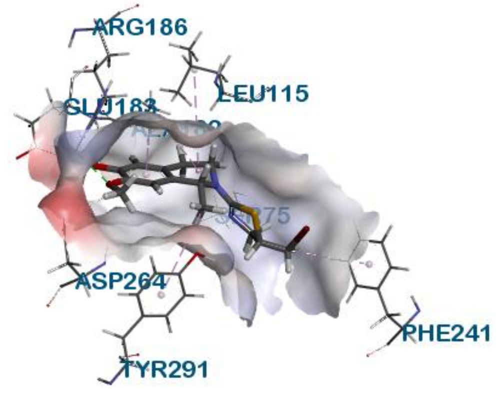

12

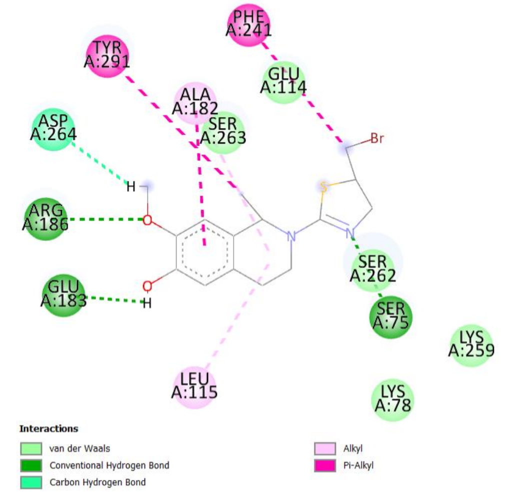

12

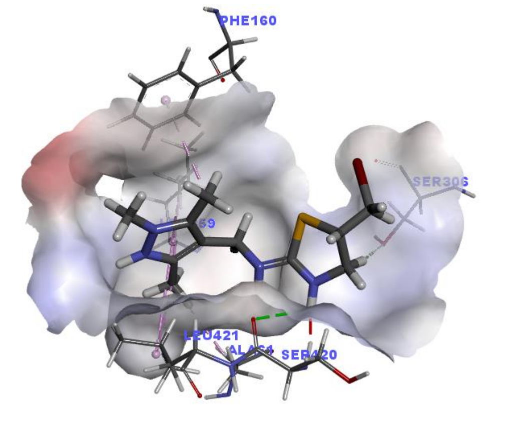
PHE160
SER306
LEU421
SER420

13

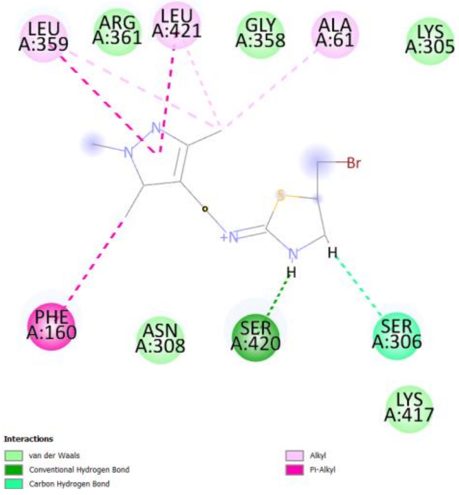
LEU A:359
ARG A:361
LEU A:421
GLY A:358
ALA A:61
LYS A:305
Br
PHE A:160
ASN A:308
SER A:420
SER A:306
LYS A:417
Interactions
van der Waals
Conventional Hydrogen Bond
Carbon Hydrogen Bond
Alkyl
Pi-Alkyl

13

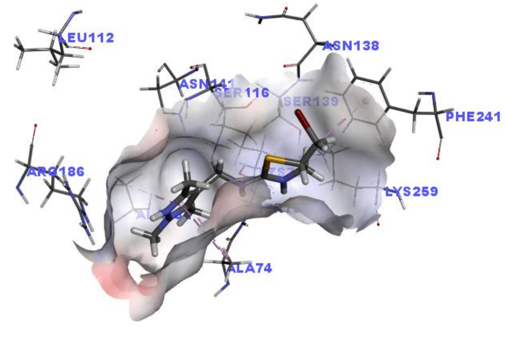
LEU112
ASN138
ASN141
SER116
SER139
PHE241
ARG186
LYS259
ALA74

13

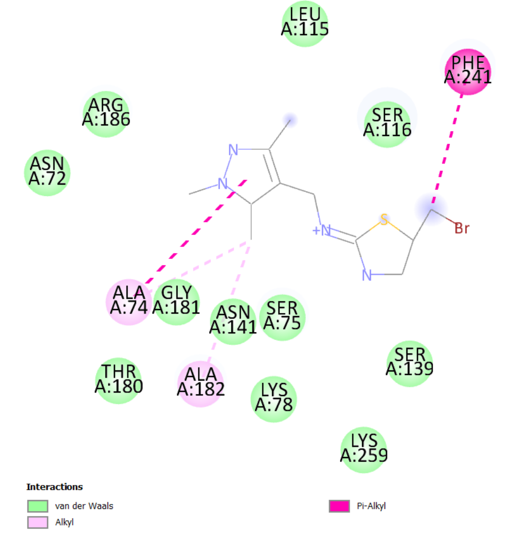
LEU A:115
PHE A:241
ARG A:186
SER A:116
ASN A:72
Br
ALA A:74
GLY A:181
ASN A:141
SER A:75
THR A:180
ALA A:182
LYS A:78
SER A:139
LYS A:259
Interactions
van der Waals
Alkyl
Pi-Alkyl

13
